# Underlying features of epigenetic aging clocks in vivo and in vitro

**DOI:** 10.1111/acel.13229

**Published:** 2020-09-15

**Authors:** Zuyun Liu, Diana Leung, Kyra Thrush, Wei Zhao, Scott Ratliff, Toshiko Tanaka, Lauren L. Schmitz, Jennifer A. Smith, Luigi Ferrucci, Morgan E. Levine

**Affiliations:** ^1^ Department of Pathology Yale University School of Medicine New Haven Connecticut USA; ^2^ Department of Big Data in Health Science, School of Public Health and the Second Affiliated Hospital Zhejiang University School of Medicine Hangzhou China; ^3^ Department of Epidemiology School of Public Health University of Michigan Ann Arbor Michigan USA; ^4^ Longitudinal Studies Section Translational Gerontology Branch National Institute on Aging National Institutes of Health Baltimore Maryland USA; ^5^ Robert M. La Follette School of Public Affairs University of Wisconsin‐Madison Madison Wisconsin USA

**Keywords:** biological aging, cellular senescence, DNA methylation, epigenetic clock, mitochondria

## Abstract

Epigenetic clocks, developed using DNA methylation data, have been widely used to quantify biological aging in multiple tissues/cells. However, many existing epigenetic clocks are weakly correlated with each other, suggesting they may capture different biological processes. We utilize multi‐omics data from diverse human tissue/cells to identify shared features across eleven existing epigenetic clocks. Despite the striking lack of overlap in CpGs, multi‐omics analysis suggested five clocks (Horvath1, Horvath2, Levine, Hannum, and Lin) share transcriptional associations conserved across purified CD14+ monocytes and dorsolateral prefrontal cortex. The pathways enriched in the shared transcriptional association suggested links between epigenetic aging and metabolism, immunity, and autophagy. Results from in vitro experiments showed that two clocks (Levine and Lin) were accelerated in accordance with two hallmarks of aging—cellular senescence and mitochondrial dysfunction. Finally, using multi‐tissue data to deconstruct the epigenetic clock signals, we developed a meta‐clock that demonstrated improved prediction for mortality and robustly related to hallmarks of aging in vitro than single clocks.

## Graphical abstract

We compared 11 existing epigenetic clocks on the basis of their functional characteristics, transcriptional associations, and ability to capture hallmarks of aging. We then decomposed their signals and recombined them into a “meta‐clock.” This meta‐clock showed stronger prediction of all‐cause mortality than any one epigenetic clock and was able to distinguish tumor from normal tissue and capture epigenetic changes in two types of senescence (replicative and oncogene induced).

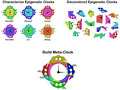

## 1 INTRODUCTION

Developing biomarkers that can quantify or approximate biological aging holds enormous promise for both basic and translational biomedical research (Kennedy et al., 2014; Lopez‐Otin, Blasco, Partridge, Serrano, & Kroemer, 2013; Sierra & Kohanski, 2017). One of the most promising biomarkers of aging that has emerged is the epigenetic clock. Some subset of DNA methylation (DNAm) has been shown to change predictably over the life span (Florath, Butterbach, Muller, Bewerunge‐Hudler, & Brenner, 2014; Johansson, Enroth, & Gyllensten, 2013; Rakyan et al., 2010; Teschendorff et al., 2010). Utilizing this observation, epigenetic clocks quantitatively combine DNAm levels at tens to hundreds of genomic locations into composite methylation‐based age predictors that often exhibit extremely high correlations with chronological age, upwards of *r* = 0.98 in full age range samples (a brief review of various epigenetic clocks (Table S1) can be found in Appendix S1 and other literature (Field et al., 2018)). Epigenetic clocks can also be contrasted against individuals' chronological ages to assess inter‐individual and/or inter‐tissue variability in the rate of aging (Horvath & Raj, 2018), and for a number of clocks, divergence between epigenetic and chronological age (referred to as age acceleration) has been shown to translate to differential susceptibility to death (Chen et al., 2016; Levine et al., 2018; Marioni et al., 2015; Zhang et al., 2017) and disease (Ambatipudi et al., 2017; Levine, Hosgood, et al., 2015; Levine, Lu, Bennett, & Horvath, 2015; Levine et al., 2018; Yang et al., 2016; Zheng et al., 2016).

Despite their shared theoretical interpretation, epigenetic clocks vary in their associations with health outcomes and each other. This does not seem to simply reflect a difference in the validity of the various clocks, but instead may suggest that they capture different aspects of the biological aging process, which likely stems from the differences in the outcomes and populations used to train them. For instance, most clocks were trained exclusively using samples in whole blood, whereas two clocks comprised multi‐tissue samples (Appendix S1). Additionally, while most were developed as “chronological age predictors,” three were trained using other phenotypes that reflect the effect of aging on health characteristics. The clock by Zhang et al. (2017) was developed as a predictor of all‐cause mortality; the clock by Yang et al. (2016) was trained to approximate mitotic rate; and the clock by Levine et al. (2018) was developed to approximate a multi‐system clinical aging measure that strongly correlates with age, but differentiates same‐aged individuals based on morbidity and mortality risk.

**Figure 1:**
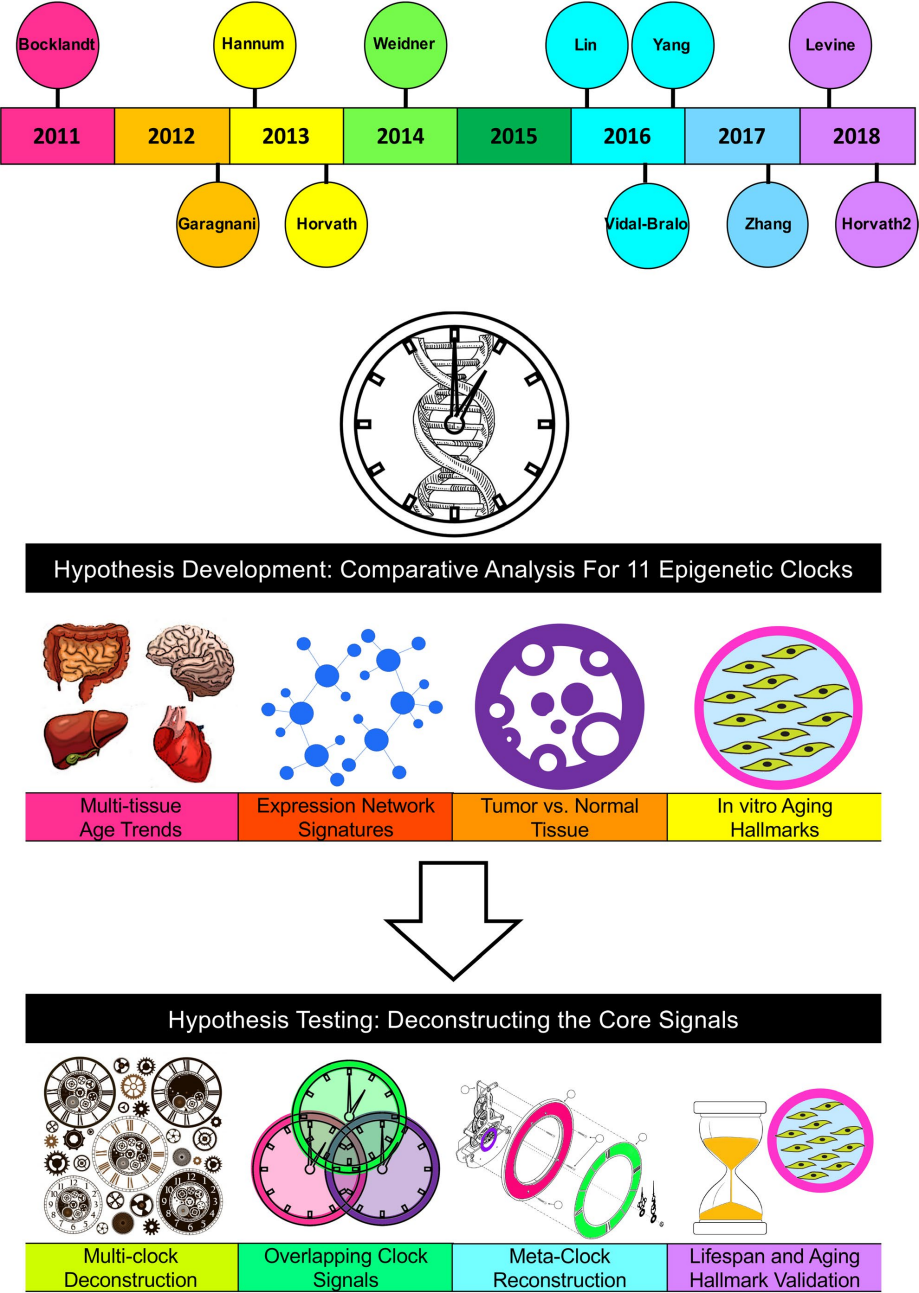
Roadmap of this comparative analysis. To simplify the description, we used the last name of the first author to refer to each clock. The upper part shows the timeline of eleven epigenetic clocks included in this study. The next two parts include hypothesis development and testing. In hypothesis development part, we did comparative analysis for eleven epigenetic clocks mainly in four aspects. In hypothesis test part, we deconstructed the core signals across them, and developed and validated a novel meta‐clock

While the clocks share both similarities and differences, we lack an understanding of the underlying processes that they capture. We hypothesized that multiple distinct epigenetic aging phenomena exist, which are captured by the various clocks to varying degrees and accuracies, and that by identifying these overlapping signals we could construct a more reliable and valid biomarker of aging. In this study, we first used multi‐omics data from diverse human tissue/cell types, coupled with in vitro experiments focusing on hallmarks of aging, to delineate a comprehensive picture of the shared and contrasting features captured across eleven existing epigenetic clocks (Figure 1). Building on these observations, we decomposed these existing epigenetic clocks and recombined their conserved features into a single “meta‐clock,” for which we demonstrated improved prediction for mortality and more robust aging associations in vivo and in vitro.

## 2 RESULTS

### 2.1 Contrasting clock characteristics across tissues/cells

The eleven epigenetic clocks considered in this study comprise 1600 CpGs, the majority of which (*n* = 1427) are specific to only one clock (Table S2). The lack of overlap in genomic locations between the clocks remains even when considering larger genomic regions (Figure S1), suggesting that clocks are not simply selecting adjacent CpGs and instead are drawing markers from entirely different regions. However, one CpG (cg09809672 in EDARADD) was included in seven of the eleven clocks. Surprisingly, the CpG (cg16867657) in ELOVL2 was only included in three clocks—Hannum, Horvath2, and Garagnani (the single CpG clock).

CpGs in the various clocks are also distinct in terms of their multi‐tissue age associations (Figure S2). Despite the fact that the Hannum clock was trained exclusively in whole blood, the CpGs comprising it show the strongest and most consistent age correlations across tissue and cell types—with approximately half of CpGs showing strong positive age correlations and the other half showing strong negative age correlations. Interestingly, CpGs in the original pan‐tissue clock by Horvath (which we refer to as Horvath1) show moderate tissue consistency, with a large proportion of CpGs exhibiting very weak to no age correlation. Given that Horvath1 was trained using 52 distinct tissues and cell types, we hypothesize that many of these non‐age‐related CpGs may actually be reflecting (and adjusting out) tissue differences. Similarly, the Levine clock also contains an abundance of CpGs with weak age correlations. The Levine clock—trained in whole blood—aimed to capture aging differences among individuals of the same chronological age. Thus, many CpGs in the Levine clock may reflect more stable differences in processes that influence aging, but do not themselves change with age in a predictable manner. Examples may include innate or acquired differences in resilience mechanisms (Levine & Crimmins, 2016), mTOR signaling (Kennedy & Lamming, 2016), inherent inflammatory/immune responsiveness (Brodin & Davis, 2017), etc.

**Figure 2:**
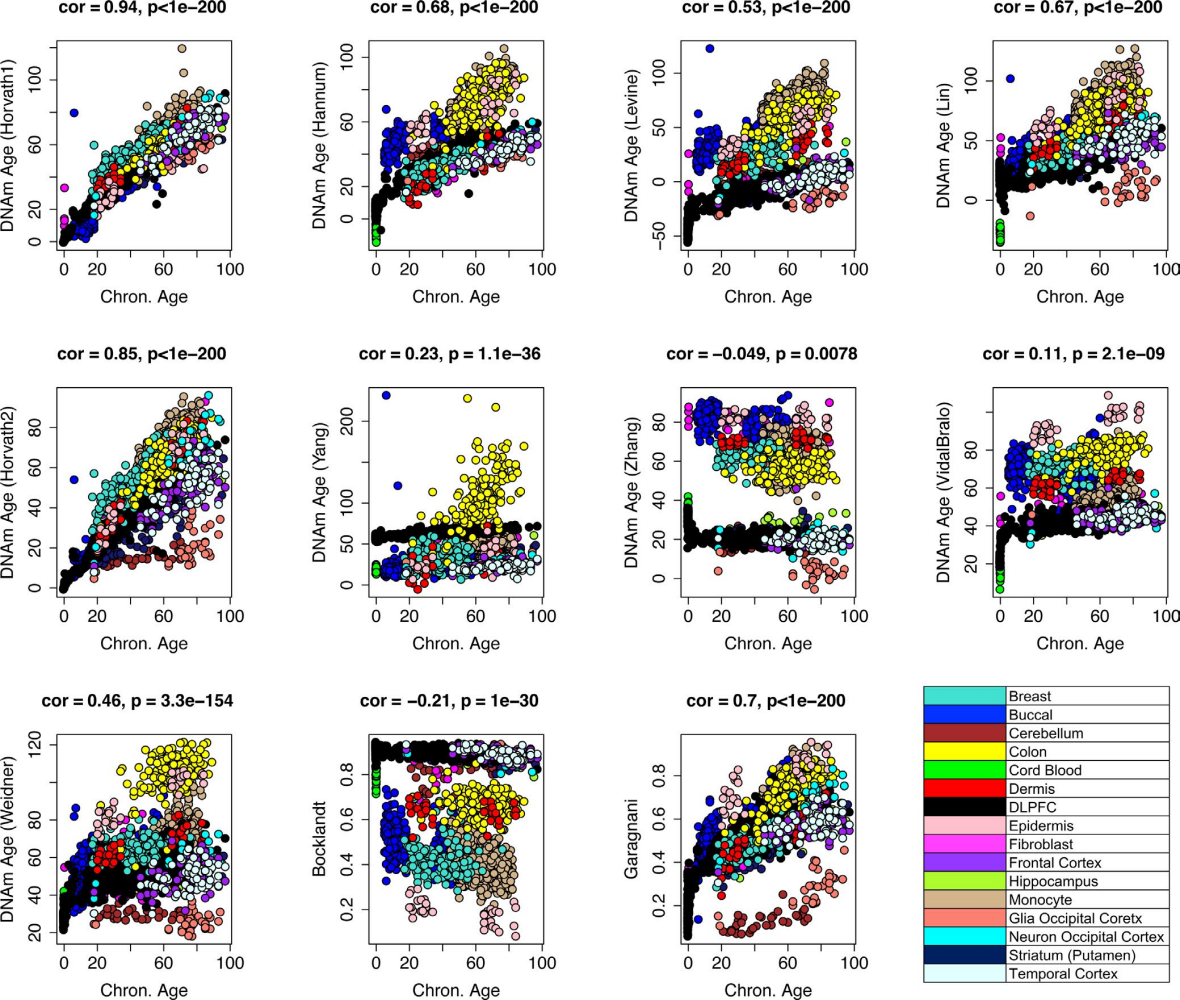
Age correlations for the clock scores across tissue and cell types. Pearson's correlations were used to assess associations between chronological age (*x*‐axis) and DNAmAge (*y*‐axis) by pooling 16 distinct tissue and cell types. Epigenetic clocks are denoted using the last name of the first author. DLPFX, dorsolateral prefrontal cortex

When considering the overall clock scores, rather than the individual CpGs that comprise them, we get a slightly different picture. As shown in Figure 2, Horvath1 exhibits the strongest age correlation across pooled tissues and cells (*r* = 0.94), which is not entirely surprising given that it was trained to do just that. This is followed by Horvath2 (*r* = 0.85), which was the only other age predictor trained on more than one tissue type. While it is often misstated that the clocks developed using DNAm from whole blood do not “work” in other tissue and cell types, we found that the clocks by Hannum, Levine, Lin, and Weidner also exhibit fairly robust multi‐tissue age correlations (*r* = 0.68, 0.53, 0.67, and 0.46, respectively). Further, the reduced correlations appear to be due to tissue differences in the slope and intercept, rather than a lack of age correlations within tissues (Figure S3). This does not suggest that epigenetic changes are tissue‐specific, but perhaps that tissues have innate differences in vulnerability to epigenetic changes that manifest as differences in the rate of epigenetic aging as a function of chronological age. Interestingly, age slopes in these clocks appear to be most distinct when comparing brain to other tissues—with brain showing a much slower increase in epigenetic age over chronological age.

### 2.2 Conserved transcriptional signature across six epigenetic clocks

**Figure 3:**
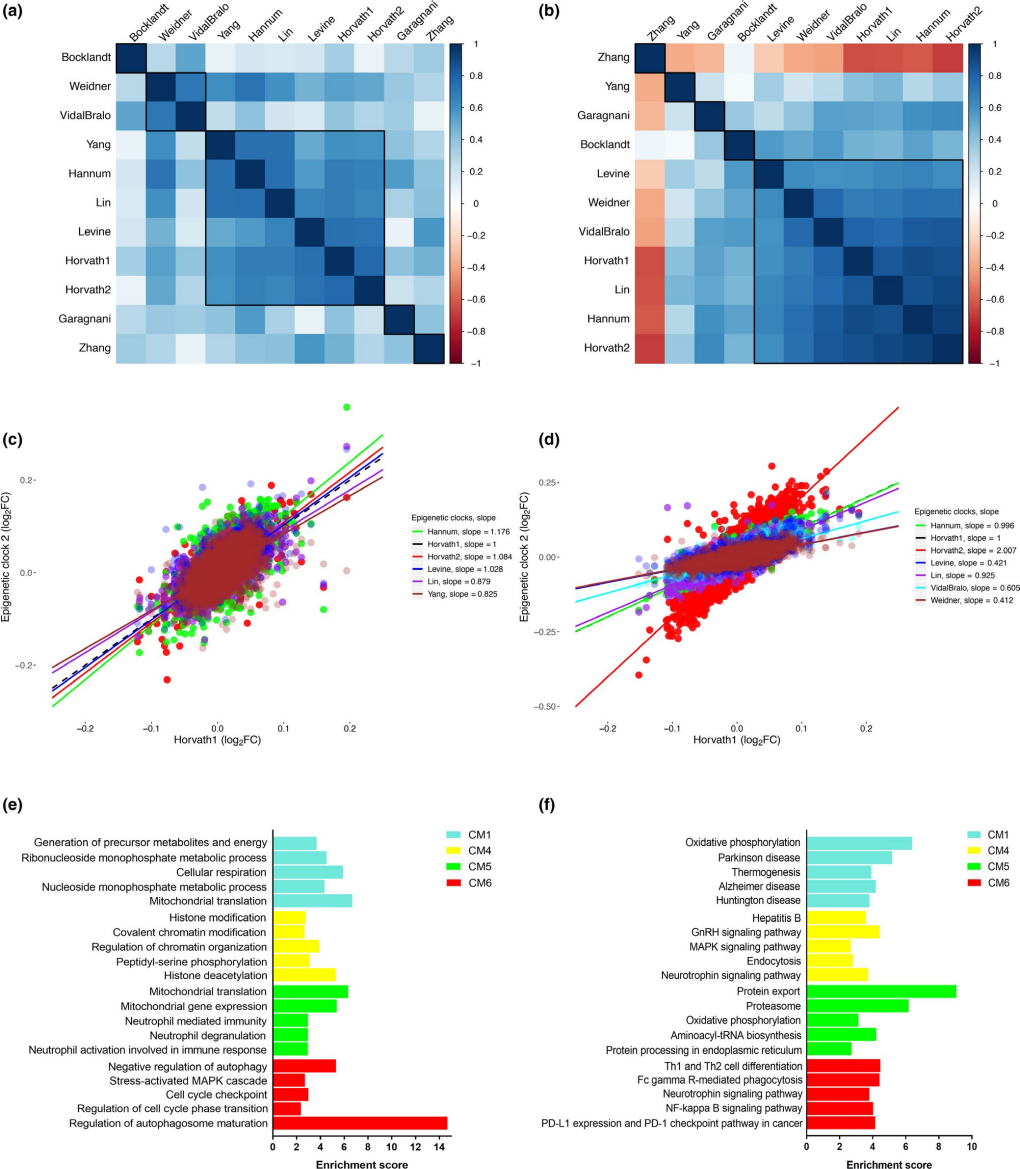
Transcriptomic pattern of 11 existing epigenetic clocks. Hieratical clustering of epigenetic clocks was performed based on the log_2_FC values for age‐adjusted associations with 8589 genes in monocytes (a) and DLPFC (b). (c) Comparisons of the strength of differential expression associations between clocks, for five clocks (reference clock = Horvath1) in monocytes. The *x*‐axis represents the log_2_FC for the association between Horvath1 and 8589 genes. The *y*‐axis represents the log_2_FC for the association between five epigenetic clocks (Yang, Hannum, Lin, Levine, and Horvath2) and 8589 genes, with clocks distinguished by colors. The slope represents the fitted line of the association between the log_2_FC for Horvath1 and the log_2_FC for each of the other five clocks, respectively. Thus, slope > 1 suggests the respective clock has stronger gene expression signals compared to Horvath1; a slope < 1 suggests Horvath1 has stronger gene expression signals compared to the respective clock; and a slope = 1 suggests comparable gene expression associations between Horvath1 and the respective clock. (d) Comparisons of the strength of differential methylation for six clocks (Levine, Weidner, VidalBralo, Hannum, Lin, and Horvath2) in DLPFC relative to Horvath1. Selected GO terms (e) and KEGG pathways (f) by the enrichment analysis for co‐expression modules (identified via WGCNA) that were shown to be associated with multiple epigenetic clocks in monocytes and/or DLPFC. Modules are denoted by color (turquoise, yellow, green, and red). For each module, the five most enriched biological processes are shown, based on *q* value (FDR). DLPFX, dorsolateral prefrontal cortex

To examine the potential functional implications of epigenetic aging assessed by various clocks, we linked the clocks to differential gene expression. As an initial step, clocks were clustered based on their shared transcriptomic expression patterns in both purified CD14+ monocytes and dorsolateral prefrontal cortex (DLPFC) using the log_2_FC values from 8589 genes as input (Figure 3a,b). Results suggested that the accelerations (i.e., chronological age‐adjusted residuals) of five clocks (Hannum, Lin, Levine, Horvath1, and Horvath2) have similar transcriptional signals in both monocytes and DLPFC. When comparing the relative levels of differential expression associated with these five clocks, we found associations to be strongest for the Hannum clock in monocytes (Figure 3c), and the Horvath2 clock in DLPFC (Figure 3d).

Next, we employed consensus weighted gene co‐expression network analysis (WGCNA), to identify gene co‐expression modules shared across monocytes and DLPFC. Modules can be thought of as tightly clustered genes that appear to operate as a network. We identified 16 co‐consensus expression modules (CM, Figure S4) shared in monocytes and DLPFC. We then calculated a summary value for each module, known as the eigengene, and tested the associations between module eigengenes and epigenetic clocks (accounting for age). Several module eigengenes displayed robust and consistent clock correlations across the two tissues (Figure S5). The co‐expression modules with the most robust clock associations after adjusting for age were the turquoise module (inverse association), green module (inverse association), yellow module (positive association), and red module (positive association). We then performed Gene Ontology (GO) enrichment analysis using the genes assigned to each of these modules, individually. We find that the turquoise module is enriched for genes involved in “cellular respiration” and “mitochondrial translation,” suggesting that epigenetic aging is associated with decreased transcription for genes involved in these biological processes. The green module, for which decreased transcription was also associated with epigenetic clocks was enriched for genes involved in “neutrophil‐mediated immunity.” Two modules for which increased expression was associated with epigenetic aging were the yellow module and the red module. The yellow module was enriched for genes involved in ‘chromatin organization’ and “histone modifications,” while the red module was enriched for genes involved in “regulation of autophagosome maturation” and “negative regulation of autophagy” (Figure 3e). Complete results for associations of eigengenes with epigenetic clocks, as well as module‐specific GO and KEGG enrichment are available in Tables S3 and S4.

### 2.3 Epigenetic clocks distinguish cancer vs normal tissues

**Figure 4:**
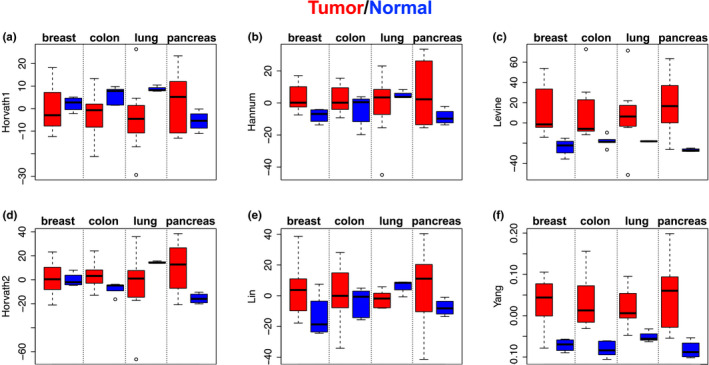
Epigenetic clocks distinguish cancer vs. normal tissues. DNAmAge (adjusted for tissue type and age of the donor) was compared between tumor (red color) and normal tissue (blue color) for breast, colon, lung, and pancreas. Bars indicate standard errors

Given the link between epigenetics aging and cancer (Ambatipudi et al., 2017; Levine, Hosgood, et al., 2015; Teschendorff et al., 2010; Yang et al., 2016; Zheng et al., 2016), we examined whether epigenetic clocks differ in terms of distinguishing tumor vs. normal tissues using data (GSE53051) from four different tumor/tissue types (breast, colon, lung, and pancreas cancer) (Timp et al., 2014). Results (Figure 4) showed that tumor samples had accelerated epigenetic aging relative to normal tissue using two (Levine and Yang) of the six epigenetic clocks shown to have conserved transcriptional signals in monocytes. Hannum showed slight acceleration in tumor vs normal for all tissues, except lung, while the other three clocks did not show consistent patterning for tumor vs. normal samples. The findings were replicable in two independent datasets for breast and colon cancer (Table S5).

### 2.4 In vitro evidence linking epigenetic clocks, cellular senescence, and mitochondrial depletion

**Figure 5:**
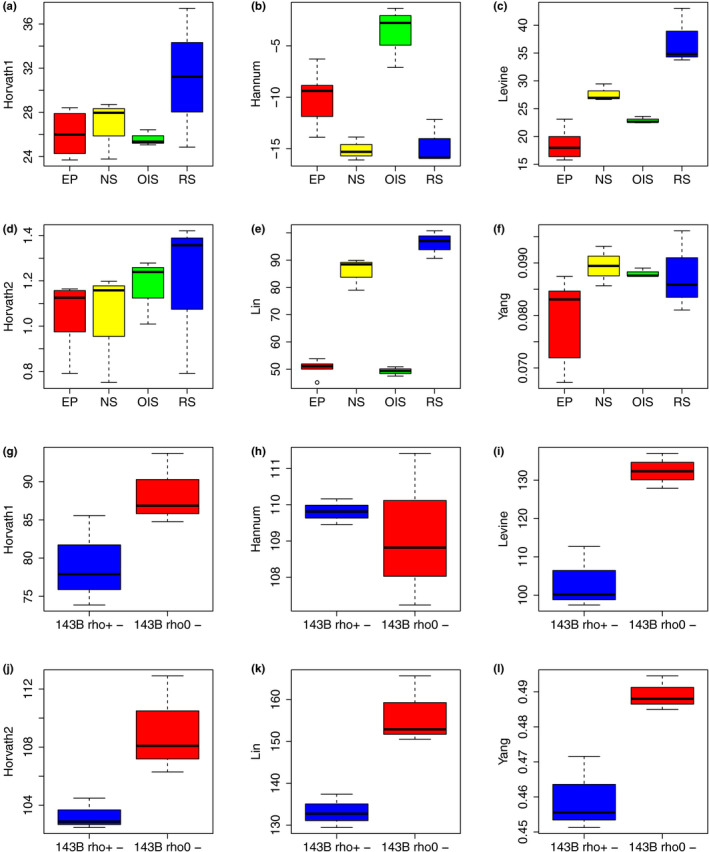
Epigenetic clocks, cellular senescence, and mitochondrial DNA depletion. (a–f) DNAmAge was estimated in BJ fibroblasts using early passage (EP) control samples, near senescent cells (NS), terminally passaged replicative senescent cells (RS), and oncogene‐induced senescent (OIS) cells via HRAS. (g–l) DNAmAge was estimated and compared between control (rho+) and mitochondrial DNA depleted (rho−) 143B cells. Bars indicate standard errors

To link epigenetic clocks to two well‐known hallmarks of aging—cellular senescence and mitochondrial dysfunction—we examined DNAm data from both cultured fibroblasts (GSE91069) and 143B cells (GSE100249). Results showed (Figure 5a–f) that the clocks by Lin and Levine displayed a sequential increase as cells transition from early passage (EP) to near senescent (NS), and finally to replicative senescence (RS)—NS vs. EP (*p*‐value = 6.8E−8, 1.3E−4, and 9.7E−4, respectively), and RS vs. EP (*p*‐value = 4.5E−9, 6.5E−7, and 1.9E−6, respectively). Of these, only Levine showed a suggestive increase when comparing oncogene‐induced senescence (OIS) vs. EP (*p* = 0.056). Conversely, the only clock to show significant increase in OIS vs. EP was Hannum (*p* = 4.0E−3); however, paradoxically, Hannum suggested RS and NS had lower epigenetic acceleration relative to EP.

Given the enrichment for mitochondrial processes observed in our co‐expression modules of interest, we examined the relationship between mitochondrial DNA (mtDNA) depletion and epigenetic clocks using in vitro DNAm data from 143B cells [30]. Clock scores were estimated and then compared between 143B cells with chronically depleted mtDNA (rho0) and 143B controls (Figure 5g–l), using three independent biological replicates for each. Linear regression revealed that rho0 cells had increased epigenetic ages according to Levine (*p* = 0.005), Lin (*p* = 0.012), and Yang (*p* = 0.012). A slightly weaker increase was also observed for Horvath2 (*p* = 0.048).

### 2.5 Identify shared central signals by decomposing epigenetic clock and re‐assembling into a novel “meta‐clock”

Our results up to this point suggested the epigenetic clocks captured both convergent and divergent signals, which was reflected in their patterning with age, associations with transcriptional signatures, age‐related outcomes, and response to in vitro conditions. To identify the shared signals, we used data from diverse tissues with diverse cell types and compositions (whole blood, DLPFC, epidermis/dermis, and breast) to cluster the clock CpGs into co‐methylation modules/networks. Using consensus WGCNA, we identified 15 co‐methylation modules containing 878 CpGs out of the 1600 clock CpGs (Figure S6).

Next, we compared the proportions of CpGs in the 15 modules across the various clocks. While most clocks differed, Horvath1 and Levine appeared to be composed of similar distributions of module CpGs. However, even though they may include similar numbers, the signals captured by CpGs in the modules may be differentially weighted when it comes to the overall clock scores.

To test this, we borrowed the weights from the original clock calculations to estimate the portion of that clock (subclocks) captured by each module. For example, the turquoise subclock for Horvath1 was calculated by setting all CpGs not in the turquoise module to 0 and then calculating the Horvath1 clock in accordance with published methods. This produced a score that represents the portion of the Horvath1 clock that turquoise CpGs were responsible for. Repeating this for all module‐clock combinations produced a total of 85 subclocks—16 modules by six clocks, with some missing pairs given that not all clocks contained CpGs from all modules.

To test whether the module CpGs were being similarly weighted across the clocks, we examined the associations between subclocks within each module. In doing so, we found that for about half of the modules, clocks were estimating the same, or similar, signals (Figure S7), whereas for the other half, the signals were often inversely weighted, for example, in some clocks they confer accelerated aging, while in others they signal decelerated aging.

**Figure 6:**
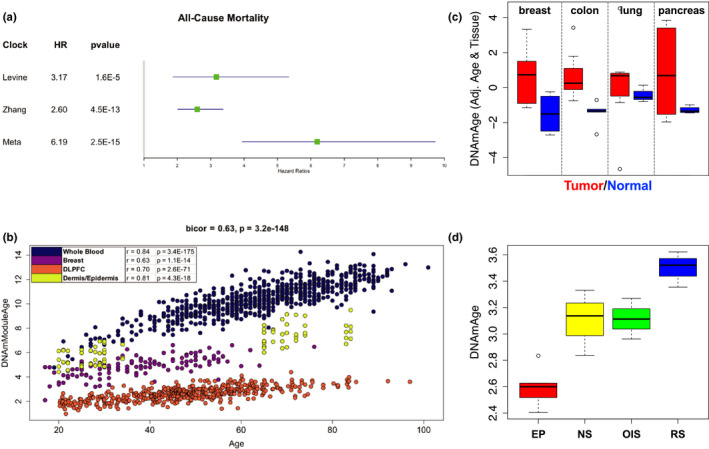
Meta‐clock validation. (a) Results for mortality prediction using the validation sample from the Framingham Heart Study (FHS), in comparison with two robust mortality prediction clocks. Bars represent 95% confidence intervals for hazard ratios (green point). (b) Biweight midcorrelations between the meta‐clock and chronological age when pooling four tissues, denoted by color. Tissue‐specific biweight midcorrelations and *p*‐values are shown in the legend. (c) Meta‐clock estimates comparing tumor (red) versus normal (blue) tissue across four cancer/tissue types (breast, colon, lung, and pancreas). Bars indicate standard errors. (d) Meta‐clock estimates in BJ fibroblasts, comparing early passage (EP) control samples, near senescent cells (NS), terminally passaged replicative senescent cells (RS), and oncogene‐induced senescent (OIS) cells via HRAS. Bars indicate standard errors. DLPFC, dorsolateral prefrontal cortex

How to best combine different epigenetic aging phenomenon to predict chronological vs. biological age is currently unknown. Additionally, it is likely that each of the clocks has both weaknesses and strengths and that combining the best “parts” across them may produce a better overall aging measure. To test this, we used data from the Framingham Heart Study (FHS) to rebuild a new aging predictor. Elastic net cox regression was used to train an all‐cause mortality predictor (*n* = 2993, with 256 deaths) using the 85 subclocks as input. Of these, fourteen were selected by the model for inclusion in what we call the “meta‐clock” (Table S6). Using an independent validation sample (*n* = 943, with 63 deaths), the meta‐clock was found to be a better mortality predictor than all the original clock scores (Figure 6a). For instance, the meta‐clock had a standardized hazard ratios (HRs) of 6.19 (*p* = 2.5E−15), higher than the two best mortality predictors considered herein—Levine (HR, 3.17, *p* = 1.61E−5) and Zhang clock (HR, 2.60, *p* = 4.5E−13). Another epigenetic age measure, GrimAge, has been shown to be a robust mortality predictor and should be compared. However, because GrimAge was trained in the FHS cohort, we were not able to conduct a comparison using independent data that would not be over‐fit.

To further validate the meta‐clock, we repeated the above comparative analysis: testing for associations with age, age‐related outcomes, and aging hallmarks (Figure 6b–d). We found that it was highly correlated with age across diverse tissues (bicor = 0.63) and exhibited higher age correlations within tissues (bicor = 0.63–0.84). It also showed deceleration of brain, similar to many of the original clocks. We observed very significant acceleration of this meta‐clock in tumor vs. normal tissue (*p* = 6.4E−11), adjusting for tissue and age of the donor. The meta‐clock is also significantly accelerated in both RS (*p* = 3.9E−5) and OIS (*p* = 1.5E−3), which was not seen for any of the original clocks. However, unlike some of the original clocks, the acceleration in rho0 cells is not significant (*p* = 0.13, Figure S8). This may be due to issues of power, given the small samples size (*n* = 6)—observation that trends were as expected. Additionally, given that the meta‐clock was optimized to predict mortality from whole blood, there may be modules that were selected that are blood or immune specific and thus may not distinguish aging hallmarks in other cell types. In moving forward, it will be important to map the specific signals captured by distinct clock modules.

Finally, using DLPFC data from the Religious Order Study and the Memory and Aging project (ROSMAP), we found that the meta‐clock is significantly associated with Alzheimer's disease (AD) neuropathology and clinical diagnosis (Table S7). This is particularly true in terms of the associations with neurofibrillary tangles (*p* = 7.0E−3) and tangle load (*p* = 7.7E−4).

## 3 DISCUSSION

Since the advent of the first epigenetic clock, new measures continue to be developed. However, there is a lack of understanding regarding how these measures compare or what they differentially capture. This is the first comprehensive comparative analysis of eleven existing epigenetic clocks, which were all developed with the fundamental purpose of capturing aging‐related alterations in the methylation landscape. Using data from diverse human tissues and cells, we compared and contrasted the clocks on the basis of their CpG characteristics, age trends, transcriptomic signals, and associations with aging hallmarks in vitro. Our data support the hypothesis that despite their distinctions, there exists a core signal captured across many of them.

Results suggested that many of the epigenetic clocks are capturing shared transcriptional signal/s, some of which have been implicated in aging. For instance, in both purified monocytes and DLPFC decreased expression of metabolism and/or immunity‐related genes, and increased expression of genes involved in chromatin modifications and/or negative regulation of autophagy were generally associated with higher epigenetic age across clocks. The links between mitochondrial function and aging were described more than half a century ago (Rockstein & Brandt, 1963), and mitochondrial dysfunction is still considered a well‐established hallmark of aging (Kennedy et al., 2014; Lopez‐Otin et al., 2013). However, it is unclear whether epigenetic aging influences mitochondrial functioning, whether impaired mitochondria contribute to epigenetic aging, and/or whether epigenetic aging is a readout of a dysregulated system for which impaired mitochondria is a contributor. Interestingly, using an experimental in vitro model we observed that the Levine clock—and to some extent the Lin, Yang, and Horvath2 clocks—was accelerated in 143B cells with chronically depleted mtDNA (rho0). Such cells are unable to carry out oxidative phosphorylation, suggesting that the causal pathway may go from mitochondrial dysfunction → epigenetic aging.

Interesting results were also observed for an experimental model of cellular senescence in human fibroblast cultures. Building on our findings, we hypothesize that some of the signal being captured by the epigenetic clocks may reflect cellular states, such as stemness or senescent cell accumulation in various tissues with age. This is further demonstrated in another cell state—tumorigenesis, which exhibits known epigenetic modifications. As with senescence, some clocks (Yang and Levine) were clearly accelerated in tumor vs. normal adjacent tissue.

The striking difference between the clocks is the limited overlap and/or target regions for CpGs included in their calculations. This may not be surprising given the redundancy of the methylome. Our consensus network analysis results suggest that there may be regions that have either shared vulnerabilities to aging‐related changes or are involved in similar biological processes. The lack of CpG overlap may be due to the possibility that each clock randomly selects a small subset of these highly related CpGs, which may represent different pathways or hallmarks of aging.

Given the complexity of the aging process, a hypothesis supported by our results is that there exist distinct phenomena of epigenetic aging, and the use of different outcome measures, tissues, and populations in developing the clocks may influence the proportions and weights placed on each one in the construction of the overall clocks. In doing so, the clocks differentially capture various aging pathways and hallmarks, contributing to the lack of correlation and differential associations with aging outcomes. By deconstructing the clocks into submodules and recombining them into a more robust epigenetic aging measure, we showed that in comparison with the original eleven clocks, this new clock is significantly better at predicting mortality risk based on DNAm in blood, and also better captures aging cell states, such as tumorigenesis and senescence via multiple diverse inducers. This new clock also tracks with age in various tissues and relates to tissue‐specific aging outcomes, such as neurodegeneration in brain—above and beyond what is captured by chronological age alone.

In summary, our results suggest that while clocks differ in their CpG components and associations, there are core signals across them, which, when modeled, produce a more robust epigenetic aging measure. These findings are a first step in uncovering the underlying biology of epigenetic clocks, which will facilitate the development of more reliable and valid biomarkers of aging for clinical and translational research, but more importantly will be essential for discovering the drivers of aging and developing interventions to target them.

## 4 EXPERIMENTAL PROCEDURES

Experimental procedures including data analysis are described in the Appendix S1.

## CONFLICT OF INTEREST

The authors declare no competing interests.

## AUTHOR CONTRIBUTIONS

ML conceived the project and study design. ZL, ML, DL, KT, SR, and WZ performed the data analysis. ZL and ML drafted the manuscript. All authors revised and approved the manuscript.

## Data Availability

Raw data and processed data are available at Gene Expression Omnibus (https://www.ncbi.nlm.nih.gov/geo), dbGaP, or AMP‐AD Knowledge Portal, as detailed in the Appendix S1.
