# Supplementary material for: Underlying features of epigenetic aging clocks in vivo and in vitro
Source: Aging Cell. 2020 Sep 15;19(10):e13229. doi: 10.1111/acel.13229 (PMC7576259; doi:10.1111/acel.13229)
Supplement: Supplementary file 1 [file ACEL-19-e13229-s001.docx]

**SUPPORTING INFORMATION**

**Additional file 1. SI Results and Methods**

**Overview of Epigenetic Clocks**

To date, multiple epigenetic clocks have been developed for quantifying human aging (Figure 1 and Table 1). The first one was developed by Bocklandt et al (Bocklandt et al., 2011) in 2011 in saliva using data from twin pairs. A predictor including two CpGs in the promoter region of EDARADD and NPTX2 and explained over 70% of the variance in chronological age, resulting in an average accuracy (average absolute difference from observed chronological age) of about 5 years. In 2012, Garagnani et al (Garagnani et al., 2012) reported that one CpG (cg16867657) in ELOVL2 appears to be a promising biomarker of aging (r=0.92) in whole blood using data from 501 persons (9-99 years). One years later, two additional clocks were developed—one by Hannum et al (Hannum et al., 2013) and one by Horvath (Horvath, 2013)—which have since become two of the most recognized epigenetic clocks in the literature. Both the Horvath and Hannum clocks were developed using penalized regression methods (i.e., elastic net) to train a predictor of chronological age based on DNAm levels at varying numbers of CpGs throughout the human genome based on Illumina Infinium arrays (Horvath, 2013). Using 450k array data from 656 persons (19-101 years), Hannum developed an age predictor that included 71 CpGs, and shows an age correlation of 0.96 (average accuracy, 3.9) in independent validation data (Hannum et al., 2013). The clock by Horvath was developed using about 8,000 samples from 82 datasets (with either 27k or 450k Illumina arrays) that collectively incorporated 51 healthy tissues/cells, to develop a multi-tissue age predictor (often referred to as the pan-tissue clock). It includes 353 CpGs in its prediction and demonstrated similarly high age correlations of 0.97 (average accuracy, 2.9) and 0.96 (average accuracy, 3.6) in training and test datasets, respectively.

Over the years, other epigenetic clocks have been developed using slightly different methods than were used by Horvath and Hannum, including 1) quantitative and characteristic-based preselection of CpGs and 2) incorporation of other aging outcomes instead of chronological age. Two clocks have been developed by Wolfgang Wagner’s group: the 3 CpG model (Weidner et al., 2014) and the 99 CpG model (Lin & Wagner, 2015). Based on 575 pooled blood samples (27k Illumina arrays) from four different studies (0-78 years), 102 CpGs with age correlations over 0.85 were pre-selected. Weidner et al (Weidner et al., 2014) then selected 3 CpGs based on recursive feature elimination, and conduciveness in a subsequent pyrosequencing analysis, resulting in an age predictor with average accuracy of 5.4 years. This 3 CpGs model was updated for the weights using 450k DNAm array data since it was initially trained on pyrosequencing data (Lin & Wagner, 2015). Lin et al (Lin & Wagner, 2015) validated 99 CpGs out of the 102 preselected age related CpGs in 450k DNAm array, resulting in a 99 CpGs model. Similarly, Vidal-Bralo et al (Vidal-Bralo, Lopez-Golan, & Gonzalez, 2016) developed an age predictor based on 8 CpG sites, out of a preselected list of the most informative CpGs (with an age correlation over 0.85) using a training set of 390 healthy persons. Yang et al (Yang et al., 2016) developed a “mitotic clock” using 385 CpGs that met three criteria: 1) CpGs were constitutively unmethylated (or hypomethylated) in any types of fetal tissues; 2) CpGs targeted the promoters marked by the PRC2 polycomb repressive complex (also known as Polycomb group targets, PCGTs); 3) CpGs showed a trend toward hypermethylation with age. The mitotic clock is estimated as the average DNAm of the 385 CpGs and aims to capture the cellular turnover. Recently, using Illumina 450K and EPIC array data from 10 training datasets, Horvath et al (Horvath et al., 2018) developed a “skin & blood clock” based on 391 CpGs. The 391 CpGs represent those on both the 450K and EPIC platforms that also met one of two criteria: 1) high absolute correlation with chronological age in different cell types, or 2) little to no significant correlation with age. This new skin & blood clock was developed for human fibroblasts, keratinocytes, buccal cells, endothelial cells, lymphoblastoid cells, skin, blood, and saliva samples, and was shown to predict age for sorted neurons, glia, brain, liver, and even bone samples, outperforming the Horvath pan-tissue clock and Hannum clock.

In contrast, Zhang et al (Y. Zhang et al., 2017) and Levine et al (Levine et al., 2018) did not train on chronological age, but rather used age correlates that reflect morbidity and/or mortality risk. Based on replicated results (58 out of 11,063 CpGs with FDR<0.05) from an epigenome-wide association study (EWAS) for all-cause mortality, Zhang et al further selected 10 CpGs using a LASSO penalized regression method to predict all-cause mortality. Two epigenetic aging measures were then proposed—one based on continuous DNAm values of the 10 CpGs, and one based on the sum of aberrant DNAm values (defined as high-risk threshold, either the highest or lowest quartile value) of the 10 CpGs. Levine et al (Levine et al., 2018) also incorporated mortality prediction, but through a two-step process that initially involved the incorporation of clinical multi-system biomarkers. In step 1, using a Cox proportional elastic net model for aging-related mortality, Levine et al built a clinical aging measure based on 9 biomarkers (albumin, creatinine, glucose, [log] C-reactive protein [CRP], lymphocyte percent, mean cell volume, red blood cell distribution width, alkaline phosphatase, and white blood cell count) and chronological age. This novel aging measure, termed “phenotypic age” (unit in years), represented the expected age within the population that corresponds to a person’s estimated mortality risk based on clinical chemistry (Levine et al., 2018; Liu et al., 2018). In step 2, the phenotypic age variable was used as the outcome for training an epigenetic clock in whole blood using an elastic net penalized regression approach, resulting in the Levine DNAmPhenoAge, which includes 513 CpGs.

In summary, all these existing epigenetic clocks are developed starting with DNAm data for tens to hundreds of thousands of CpG sites across the genome, as measured on Illumina arrays. CpGs are then selected based on supervised machine learning or EWAS based on prediction of either chronological age or age-related outcomes. The clocks therefore incorporate a small subset of CpGs (between 1 and 513) that are differentially weighted to estimate epigenetic age.

**Table S1. Summary of recent existing 11 epigenetic clocks in human samples**

| **First Author, Year** | **Training phenotypes** | **# of CpGs** | **Tissues** | **Denotation** |
| --- | --- | --- | --- | --- |
| Bocklandt, S., 2011 | Chronological age | 1 | Saliva | Bocklandt |
| Garagnani, P., 2012 | Chronological age | 1 | Whole blood | Garagnani |
| Hannum, G., 2013 | Chronological age | 71 | Whole blood | Hannum |
| Horvath, S., 2013 | Chronological age | 353 | 51 tissues/cells | Horvath1 |
| Weidner, C.I., 2014 | Chronological age | 3 | Whole blood | Weidner^a^ |
| Lin, Q., 2016 | Mortality | 99 | Whole blood | Lin |
| Vidal-Bralo, L., 2016 | Chronological age | 8 | Whole blood | Vidal-Bralo |
| Yang Z., 2016 | Chronological age | 385 | Whole blood | Yang |
| Zhang Y., 2017 | Mortality | 10 | Whole blood | Zhang |
| Levine, M., 2018 | Phenotypic Age | 513 | Whole blood | Levine |
| Horvath, S., 2018 | Chronological age | 391 | Skin | Horvath2 |

^a^ The 3 CpGs model by Weidner et al (Weidner et al., 2014) was updated for the weights using 450k DNAm array by Lin et al (Lin & Wagner, 2015) since it was initially trained on pyrosequencing data. The updated weights were used in this study.

**RESULTS**

**Table S2: Annotations of all CpGs included in 11 the epigenetic clocks (separate CSV file)**

**Table S3. Results for the GO enrichment analysis for genes in consensus modules (separate Excel file)**

**Table S4. Results for the KEGG pathway analysis for genes in these modules (separate Excel file)**

**Table S5. Epigenetic clocks distinguish cancer vs normal tissues in two independent datasets with breast and colon cancer samples**

|  | **Beta Coefficient (p-value)** | |
| --- | --- | --- |
| **Clock** | **Breast (GSE37754)** | **CRC (GSE48684)** |
| Horvath1 | -16.78 (p=1.4e-2) | -6.64 (p=8.2e-2) |
| Hannum | 64.10 (p=6.7e-2) | 7.91 (p=1.2e-1) |
| Horvath2 | -14.66 (p=3.2e-1) | 16.91 (p=3.8e-4) |
| Levine | 148.54 (p=1.1e-2) | 36.67 (p=8.5e-6) |
| Lin | 56.42 (p=1.5e-1) | 20.69 (p=8.9e-3) |
| Yang | 0.64 (p=2.0e-4) | 0.18 (p=1.4e-13) |

CRC, colorectal cancer.

**Table S6. Selected clock modules and standardized weights for meta-clock calculation**

| **Clock Module** | **Coefficient (Stnd.)** |
| --- | --- |
| Horvath1 Cyan | 0.014025038 |
| Horvath2 Cyan | 0.026585674 |
| Horvath2 Grey | 0.069908003 |
| Horvath2 Purple | 0.110420294 |
| Levine Green | 0.036194288 |
| Levine Grey | 0.181597715 |
| Levine Pink | 0.186229681 |
| Levine Salmon | 0.003347797 |
| Hannum Brown | 0.243426214 |
| Hannum Grey | 0.268197082 |
| Hannum Purple | 0.034614119 |
| Lin Yellow | 0.168016101 |
| Zhang Grey | 0.341349658 |
| Zhang Purple | 0.090624457 |

Out of 85 clock modules, 14 were selected by penalized regression to form the novel meta-clock. They included at least one module for each of the conserved clocks, except Yang. Overall, 1 module was selected from Horvath1, 3 from Horvath2, 4 from Levine, 3 from Hannum, 1 from Lin, and 2 from Zhang.

**Table S7. Associations between meta-clock assessed in DLPFC and Alzheimer’s disease neuropathology in the ROSMAP sample**

|  | **Standardized Beta (SE)** | **P-Value** |
| --- | --- | --- |
| Amyloid | 0.100 (0.034) | 2.73E-03 |
| Neuritic Plaques | 0.072 (0.032) | 2.83E-02 |
| Diffuse Plaques | 0.056 (0.033) | 8.53E-02 |
| NFT | 0.090 (0.033) | 7.10E-03 |
| Tangle Load | 0.111 (0.033) | 7.66E-04 |
| Results Based on OLS regression, adjusting for age, sex, study, and estimated proportion of neurons. SE, standardized error. | | |

**Table S8. Results for the GO enrichment analysis for genes in proteins modules (separate Excel file)**

**Table S9. Results for the KEGG enrichment analysis for genes in proteins modules (separate Excel file)**

**Table S10. Heritability estimates of the eleven epigenetic clocks in Framingham Heart Study (separated Excel file)**

| A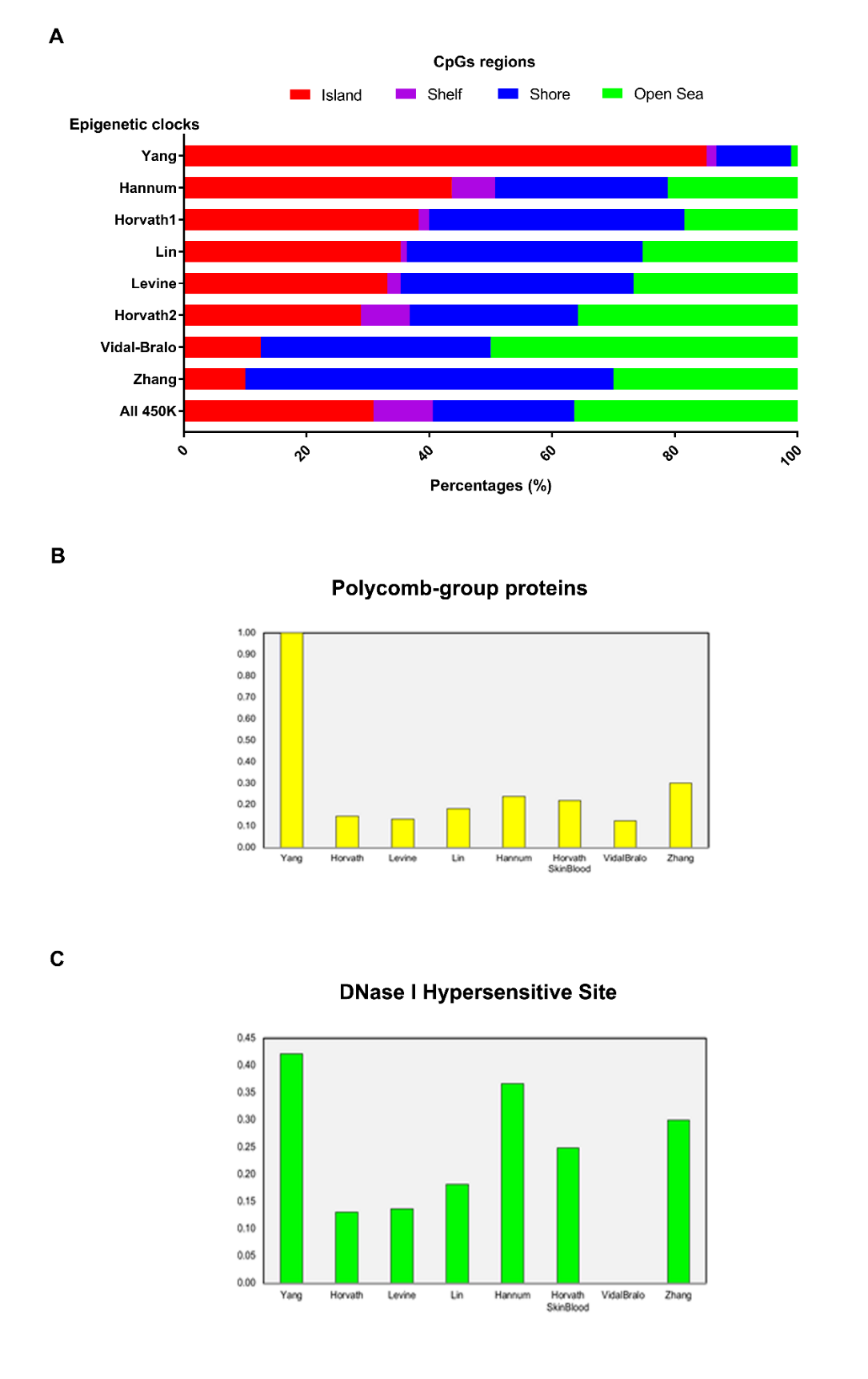 | |
| --- | --- |
| B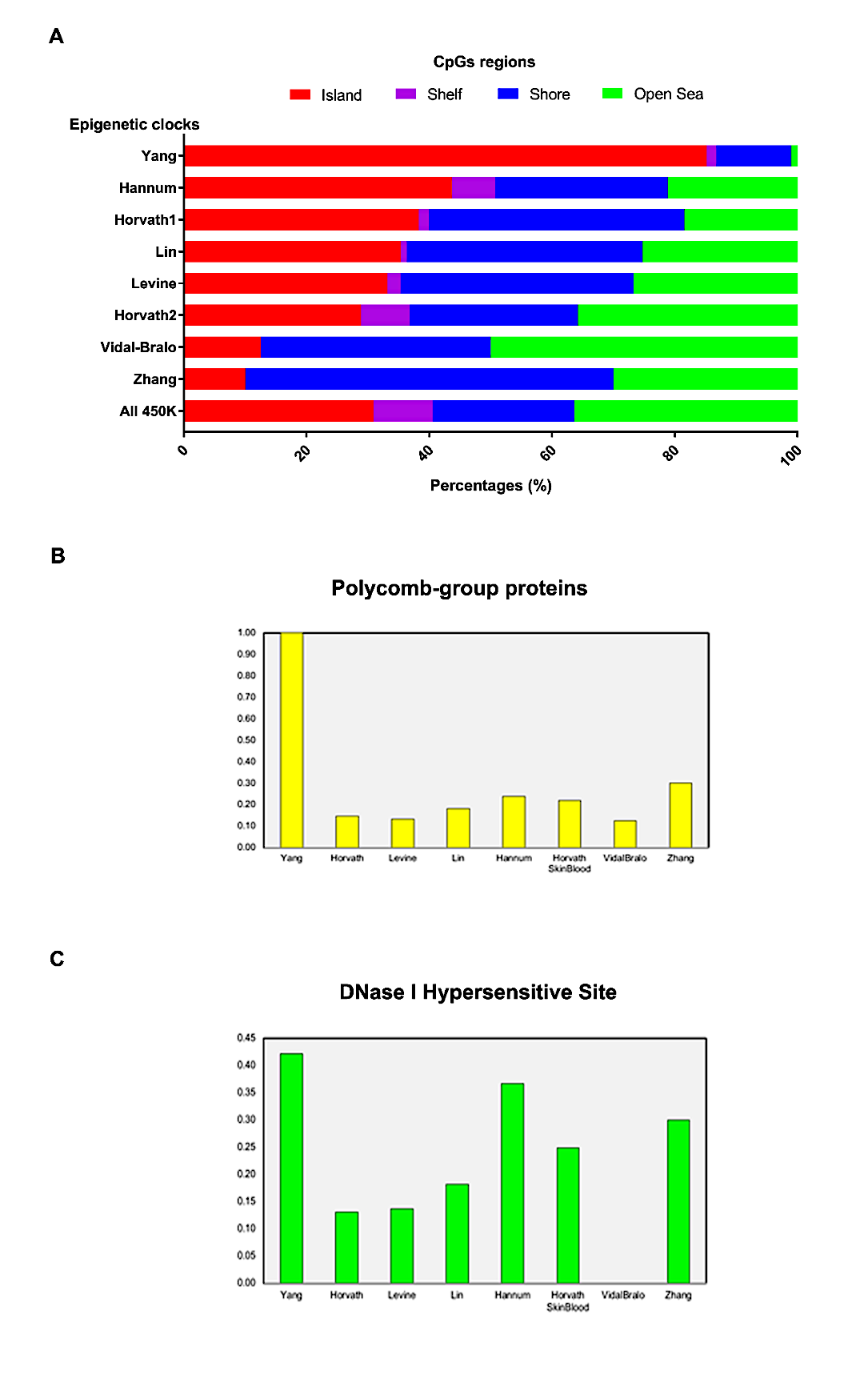 | C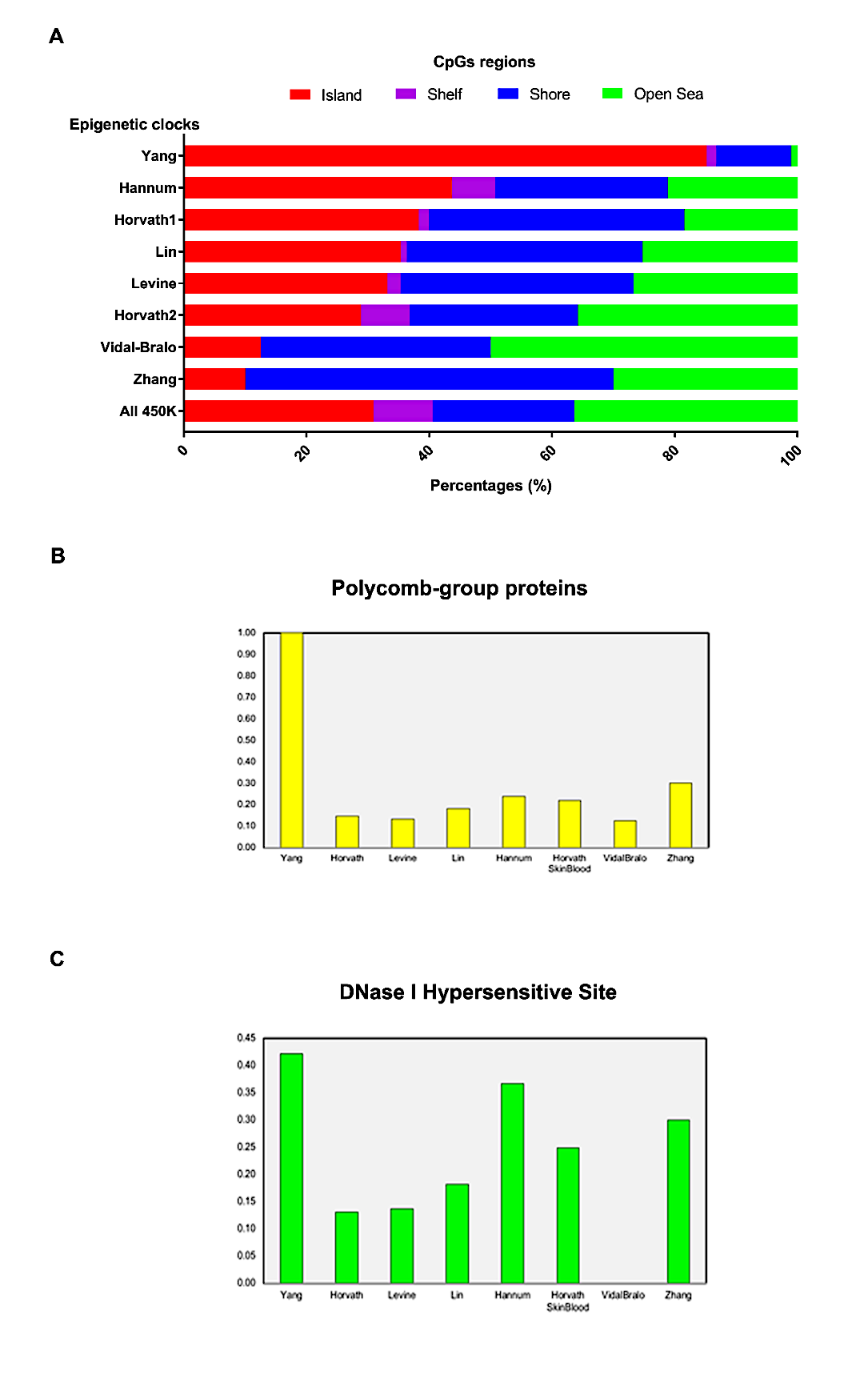 |

**Figure S1. CpG targets for those included in the 11 epigenetic clocks.** A. CpG regions for those included in the 11 epigenetic clocks. The results for Bocklandt, Garagnani, and Weidner are not presented. The single CpG in Bocklandt clock (cg09809672) is located in a Shore, while the signle CpG in Garagnani clock (cg16867657) is in an Island. One of the CpGs (cg17861230) in Weidner is located in an Island and the other two (cg02228185 and cg25809905) are in Open Seas. B. The proportions of CpGs in polycomb-group (PcG) protein targets. C. The proportions of CpGs in DNase I hypersensitive sites (DHS). For instance, Yang includes only CpGs in PcG protein targets, whereas, for the most part, CpGs in PcG protein targets make less than a third of CpGs in the other clocks (range 12-30%). Similarly, the Yang clock also has the highest proportion of DHS CpGs (42%), followed by Hannum with 37%, Zhang with 30%, Horvath2 with 25%, Lin with 18%, Levine and Horvath1 with about 13%, and both Vidal-Bralo and Weidner containing none.

**
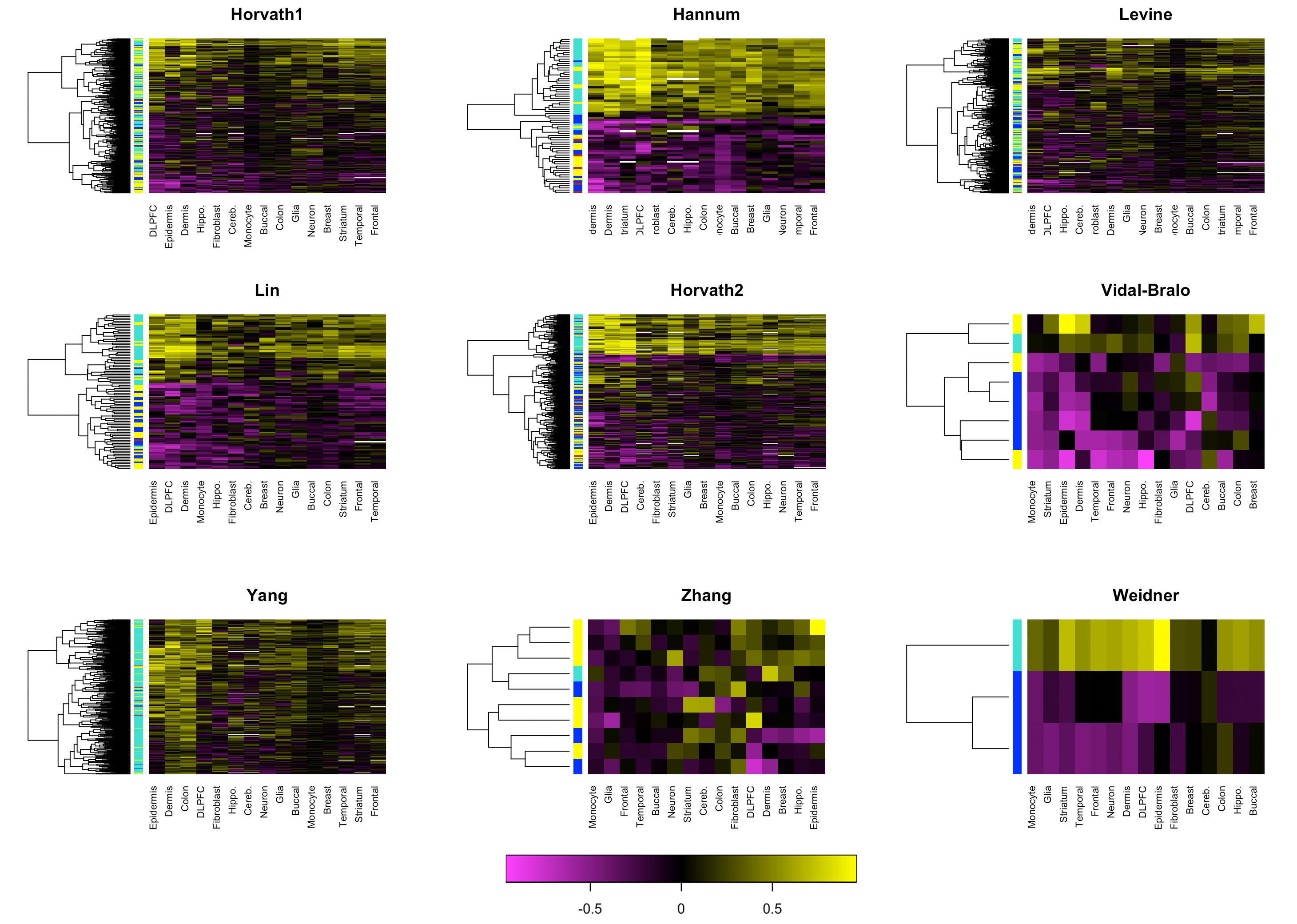
**

**Figure S2. Heatmaps (hierarchical clustering) of age correlations for the CpGs included in each epigenetic clock, across various tissues and cells.**

DLPFC dorsolateral prefrontal cortex. Y-axis colorbar reflects the type of CpG location—turquoise signifies islands, blue signifies open sea, yellow signifies shores, and brown signifies shelves.

| 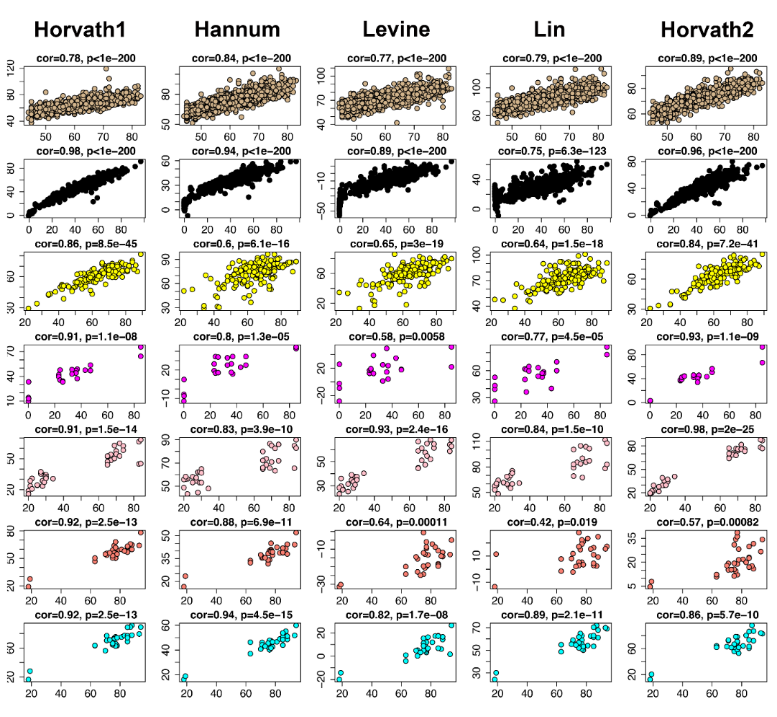 | 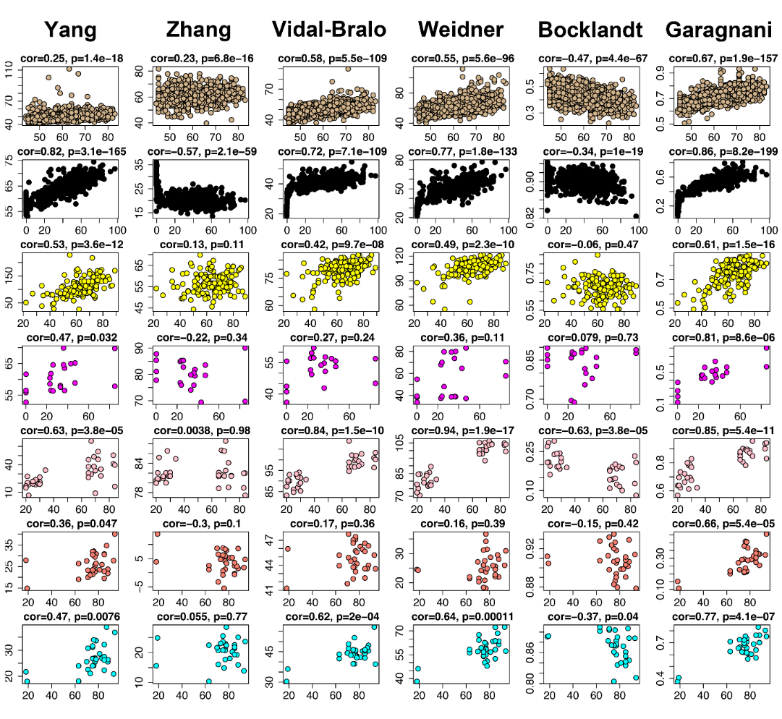 |
| --- | --- |

**Figure S3. Age correlations of the 11 epigenetic clocks in each tissue/cell.** DLPFC, dorsolateral prefrontal cortex. Starting at the top, the rows represent data from: monocytes (tan), DLPFC (black), colon (yellow), fibroblasts (magenta), epidermis (pink), glial from occipital cortex (salmon), neurons from occipital cortex (cyan). The five clocks on the right and the single CpG clock by Garagnani et al., all show robust age correlations regardless of tissue/cell type. Conversely, the Yang, Zhang, Vidal-Braol, Weidner, and Bocklandt clocks show inconsistent age correlations—with weak age prediction in at least one cell/tissue type.

|  |
| --- |
| **Figure S4.** Network dendrogram from co-expression topological overlap of 8,589 overlapped genes between purified monocytes and brain tissues (DLPFC). We identified 16 co-expression modules (Cons modules, can be thought of as tightly clustered genes that appear to operate as a network) shared in monocytes and DLPFC. |

** Figure S5. Module eigengene (consensus modules, CMs) and clock correlations and p-values in purified monocytes and brain tissues (DLPFC).** For each module, an eigengene value was calculated, representing the optimal summary score of the gene expression profile. Each cell reports the correlation (and p-value) resulting from correlating module eigengene (rows) to clock residuals, color-coded by correlation according to the color legend. For each CM, the upper shows module eigengene-clock correlations in purified monocytes, and the lower in DLPFC.

**Figure S6. Dendrogram from consensus WGCNA co-methylation analysis.** We identified 16 consensus co-methylation modules across four tissue/cell types. Colors in tope row denote module colors. The four subsequent rows illustrate the strength of age correlations for CpGs, with red representing positive (hypermethylation with age) and blue representing negative (hypomethylation with age). CpGs in the brown module show very strong positive age correlations, whereas those in the yellow module exhibit strong negative associations, particularly in blood and skin (epidermis/dermis). Those in the turquoise module have weak and/or inconsistent age correlations.


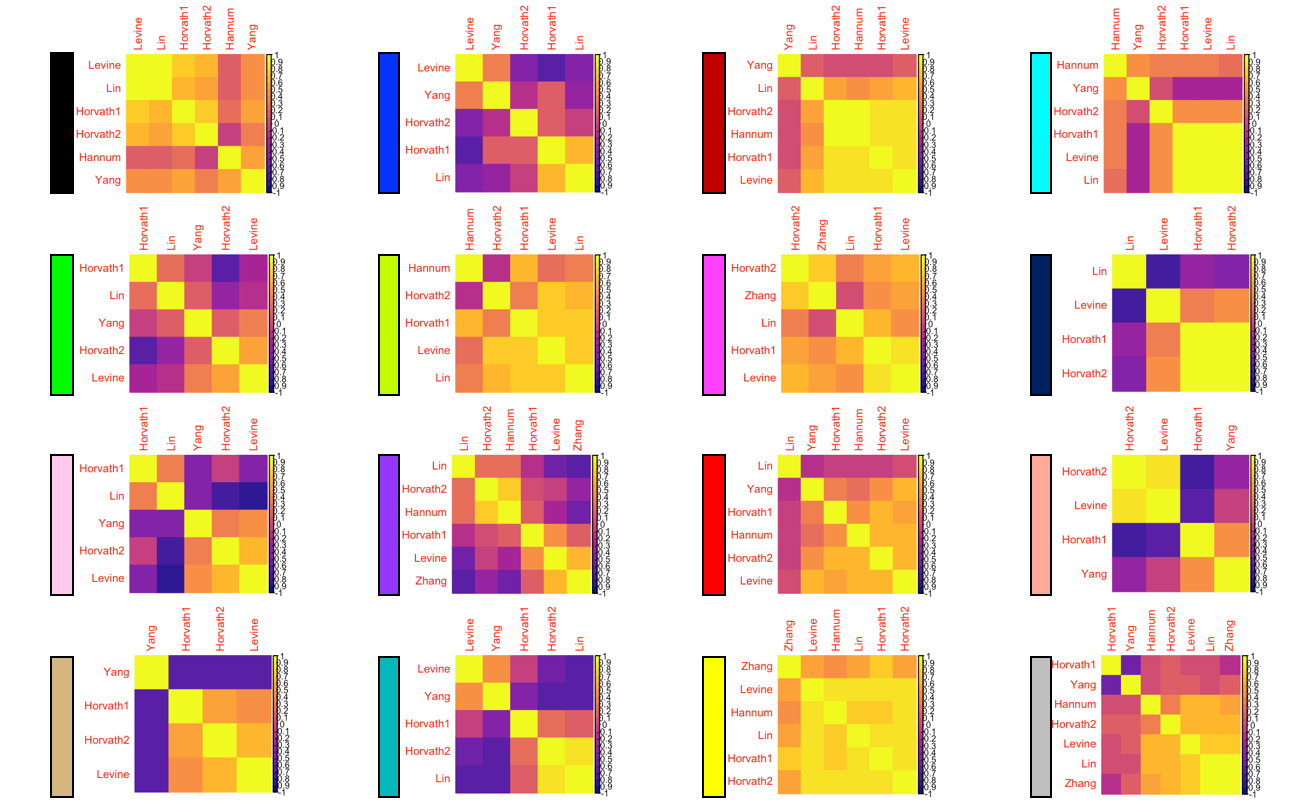


**Figure S7. Sub-Module Correlations Between Clocks.** After calculating the submodules for each clock using data from Framingham Heart Study, we analyzed module-specific correlations between the clocks in order to test whether clocks were picking-up the same signals for each module. According to the legend, colors closer to yellow denote strong positive age correlation, suggesting that clocks are capturing consistent signals. Conversely, cells colored purple denote strong negative age correlation, suggesting that one clock counts the signal towards accelerated aging, while another clock counts it towards decelerated aging.


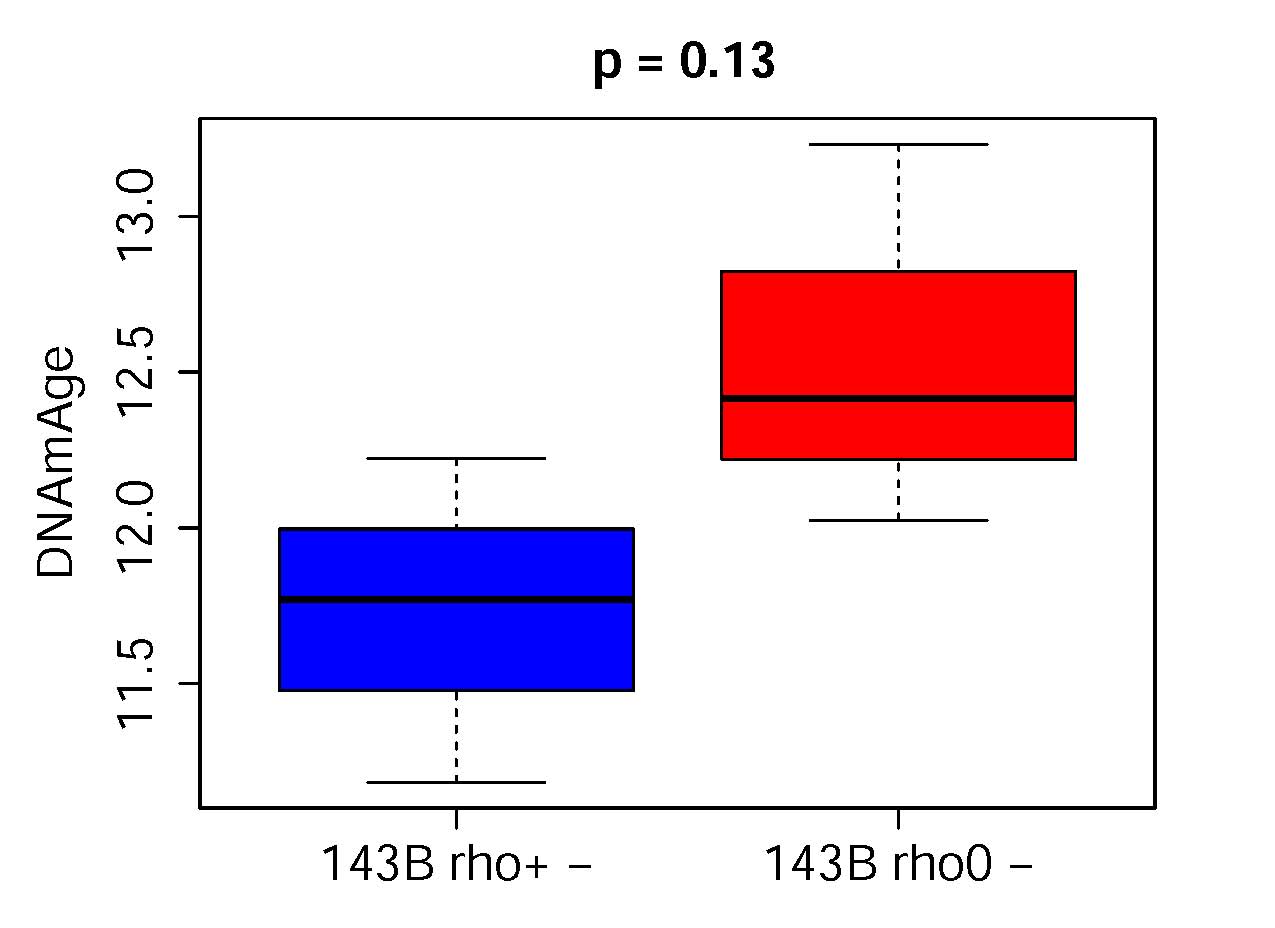


**Figure S8. In vitro evidence linking the Meta-clock and mitochondrial depletion.**

Meta-clock was estimated and then compared between 143B cells with chronically depleted mtDNA (rho0) and 143B controls, using three independent biological replicates for each. Linear regression revealed that unlike some of the original clocks, the acceleration in rho0 cells is not significant for the Meta-clock (p=0.13).


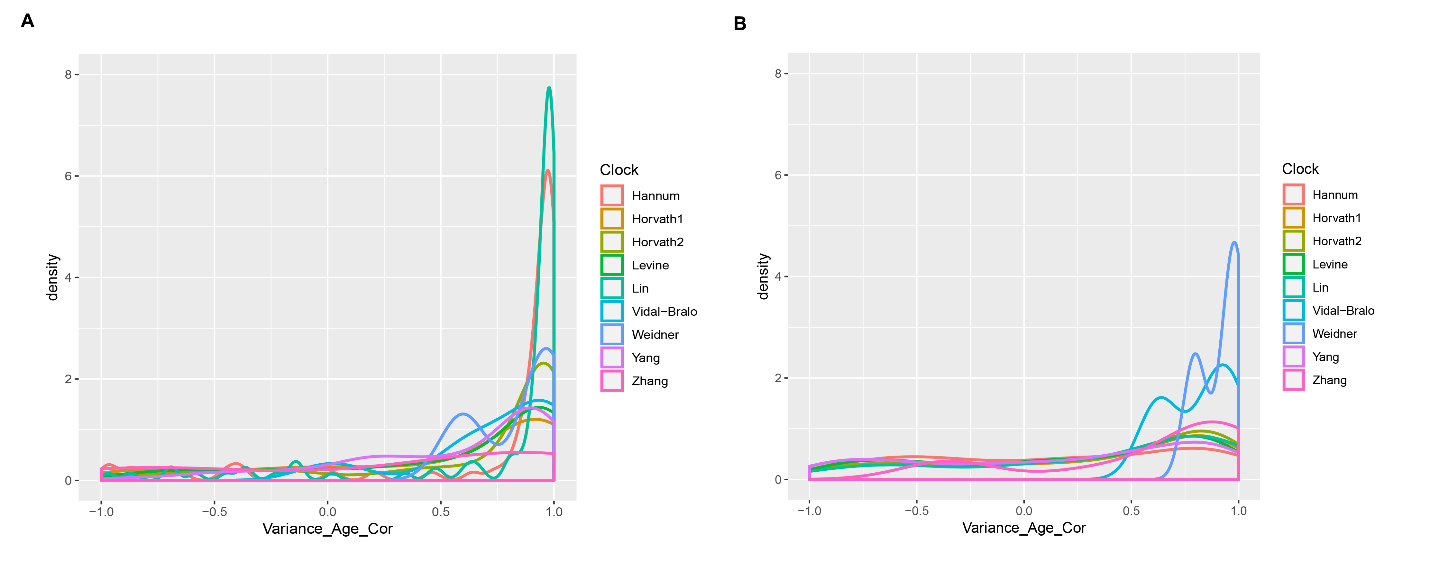


**Figure S9. Variance of each CpG using 10-year age bins in A. purified monocytes and B. brain tissue (dorsolateral prefrontal cortex (DLPFC)).** If epigenetic aging is part of an entropic process reflective of damage accumulation or dysregulation over time, one would expect between-person differences to increase with time, as people diverge in their aging trajectories. To test this, we examined the change in variance with age for each CpG. Changes in DNAm that represent random drift should exhibit increasing variance with age; conversely, DNAm changes that are more developmental should exhibit stable variance over the age range. Using data from purified CD14+ monocytes and dorsolateral prefrontal cortex (DLPFC), we estimated the correlations between the variance in CpG DNAm levels within 10-year age bins (e.g., ages 30-39, 40-49, etc.) and the midpoint for age in each bin was used when assessing the change in variance with age. Positive values suggest that variance increases with age, whereas negative values suggest variance decreases with age. Results showed that Lin and Hannum almost exclusively contain CpGs that display strong increases in DNAm variance in monocytes with age. Although to a lesser degree, the other clocks, aside from Zhang, also appear to consist of CpGs with increasing age-related variance in monocytes. Nevertheless, when examining changes in variance using data from DLPFC, the age trend is substantially reduced. In this case the Weidner (3 CpGs) and Vidal-Bralo (8 CpGs) clocks show general consensus for increasing variance with age, while the other clocks have only a slight enrichment in CpGs that exhibit increasing DNAm variance with age.

**Figure S10. The relationship between epigenetic clocks and a novel proteomic clock.** To gain additional insight into aging relationships, we examined epigenetic clock associations with a novel SOMA based plasma proteomic estimate of biological age developed by Tanaka et al (Tanaka et al., 2018). Using whole blood DNAm data from the GESTALT study (n=44) and adjusting for chronological age, we found that Levine, Hannum, and Bocklandt are positively associated with proteomic age (r > 0.2, Fig. S3 in Supplementary file 5). Conversely, Horvath1 is the only clock to exhibit an inverse association with proteomic age of (r < -0.20), suggesting that individuals who appear to be aging faster based on protein levels exhibit lower epigenetic ages in blood as assessed by the pan-tissue clock. However, given that we were underpowered, these results should be followed-up in larger samples.

| **A**  **** |
| --- |
| **B**  **** |

**Figure S11. WGCNA for 1,301 plasma proteins. (A)** We identified eight co-expression plasma protein modules (PMs, can be thought of as tightly clustered proteins that appear to operate as a network). (B) For each PM, an eigengene value was calculated. Each cell reports the correlation (and p-value) resulting from correlating module eigengene (rows) to traits clocks (adjusted for age). The table is color-coded by correlation according to the color legend.

| **A**  **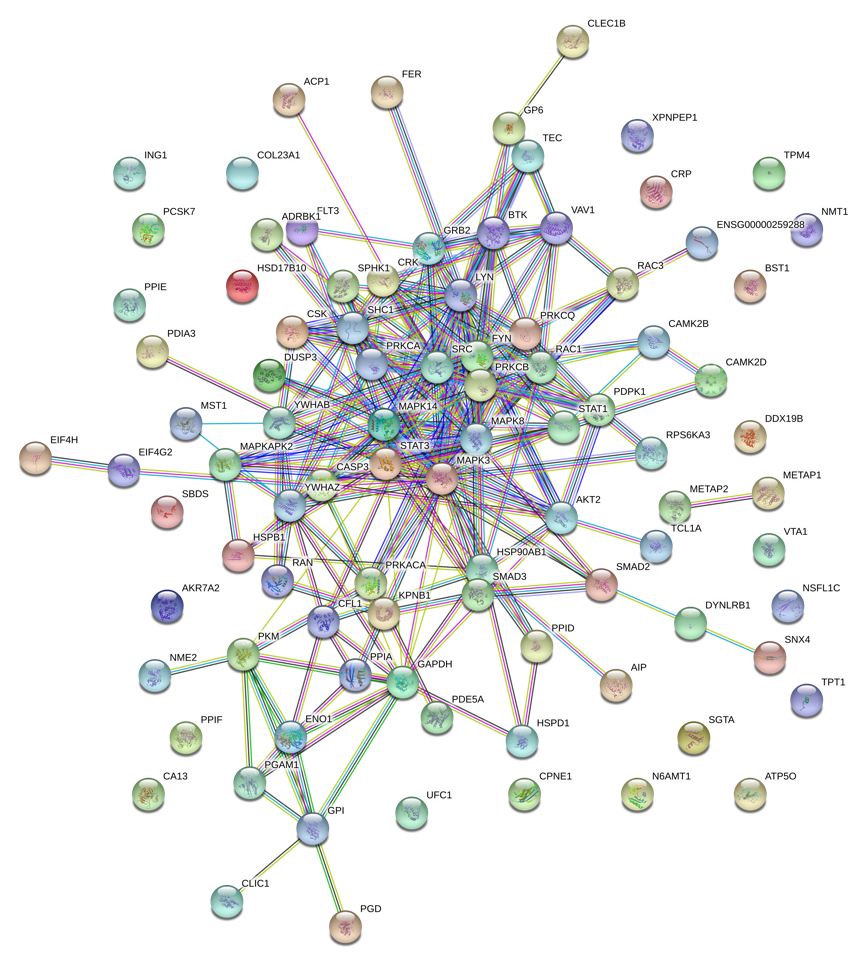** |
| --- |
| **B**  **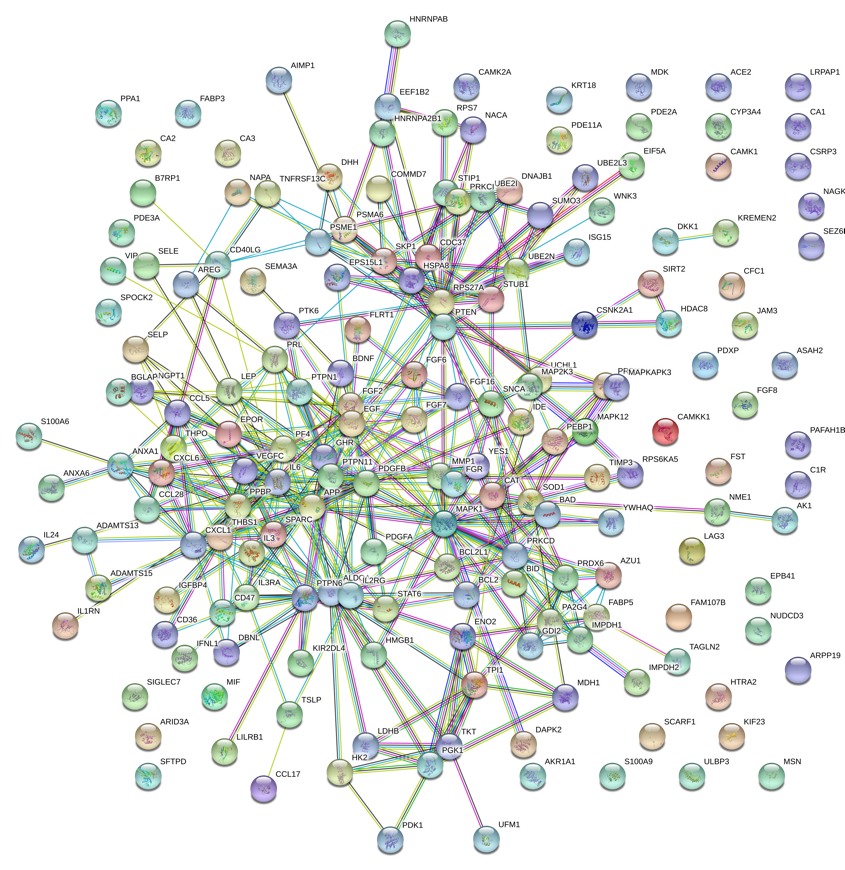** |

**Figure S12. Protein-protein interaction for select protein modules.** (A) Yellow module from plasma protein WGCNA. (B) Blue module from plasma protein WGCNA

| **A**  **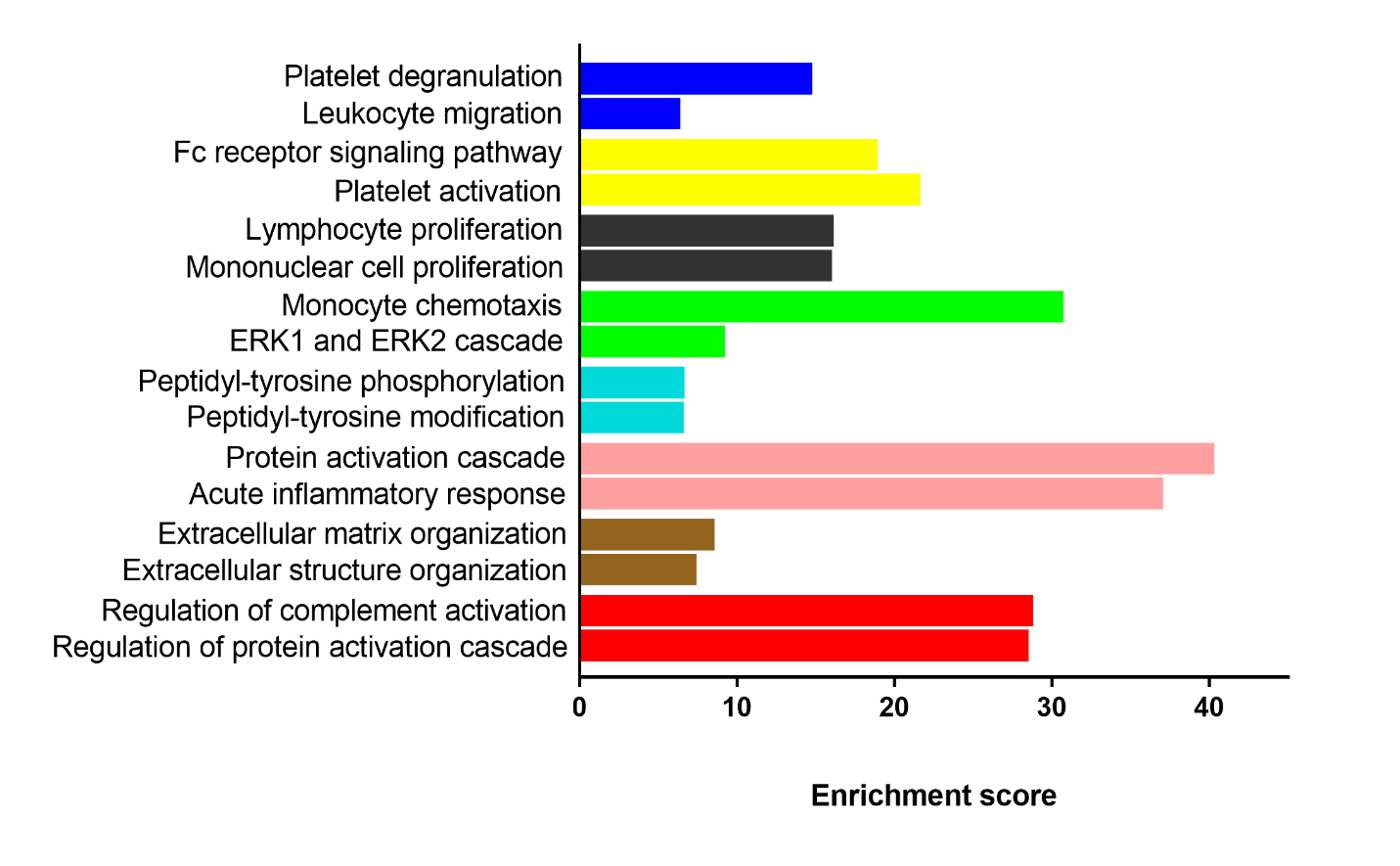** |
| --- |
| **B**  **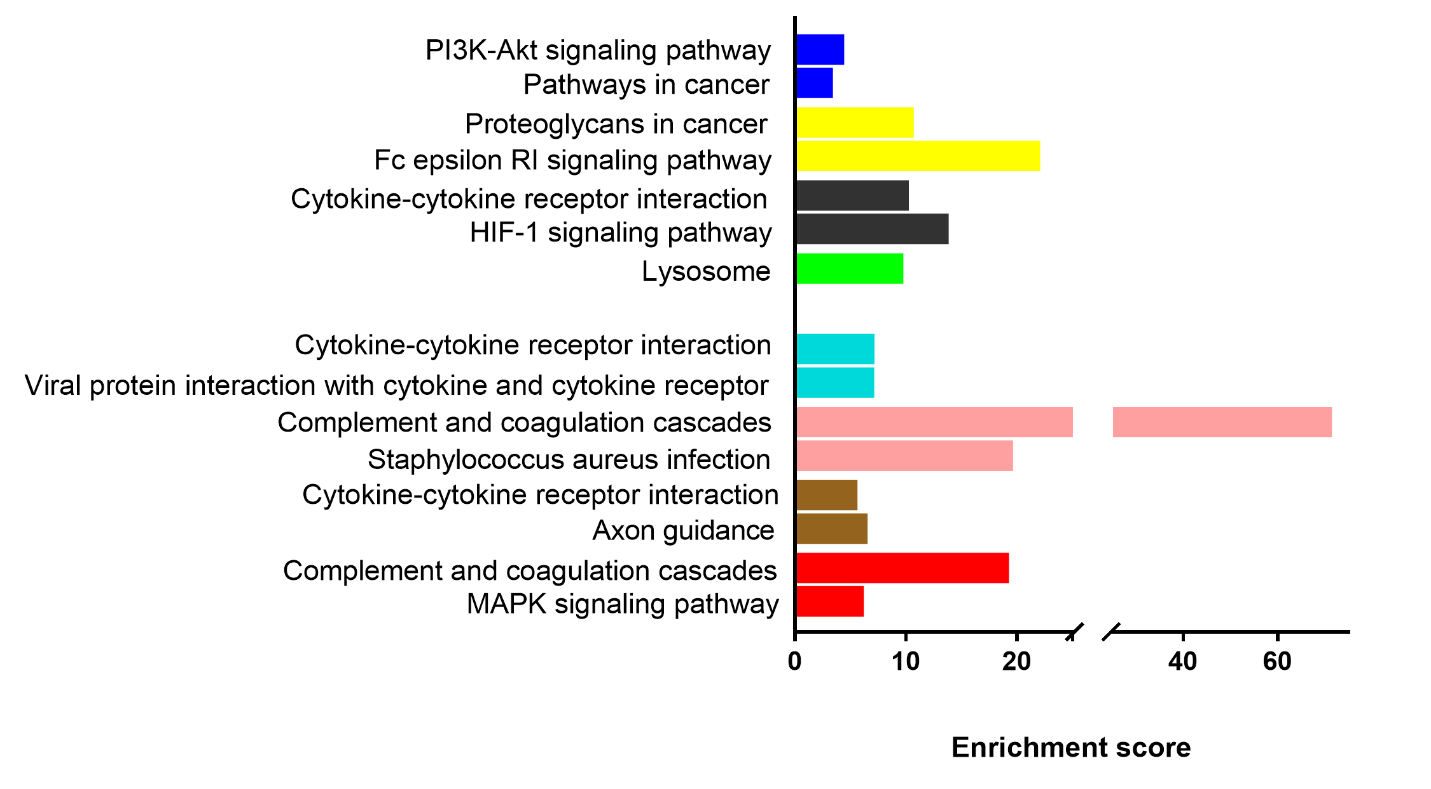** |

**Figure S13. Top two enrichment terms for genes in protein modules based on GO (A) and KEGG (B).**


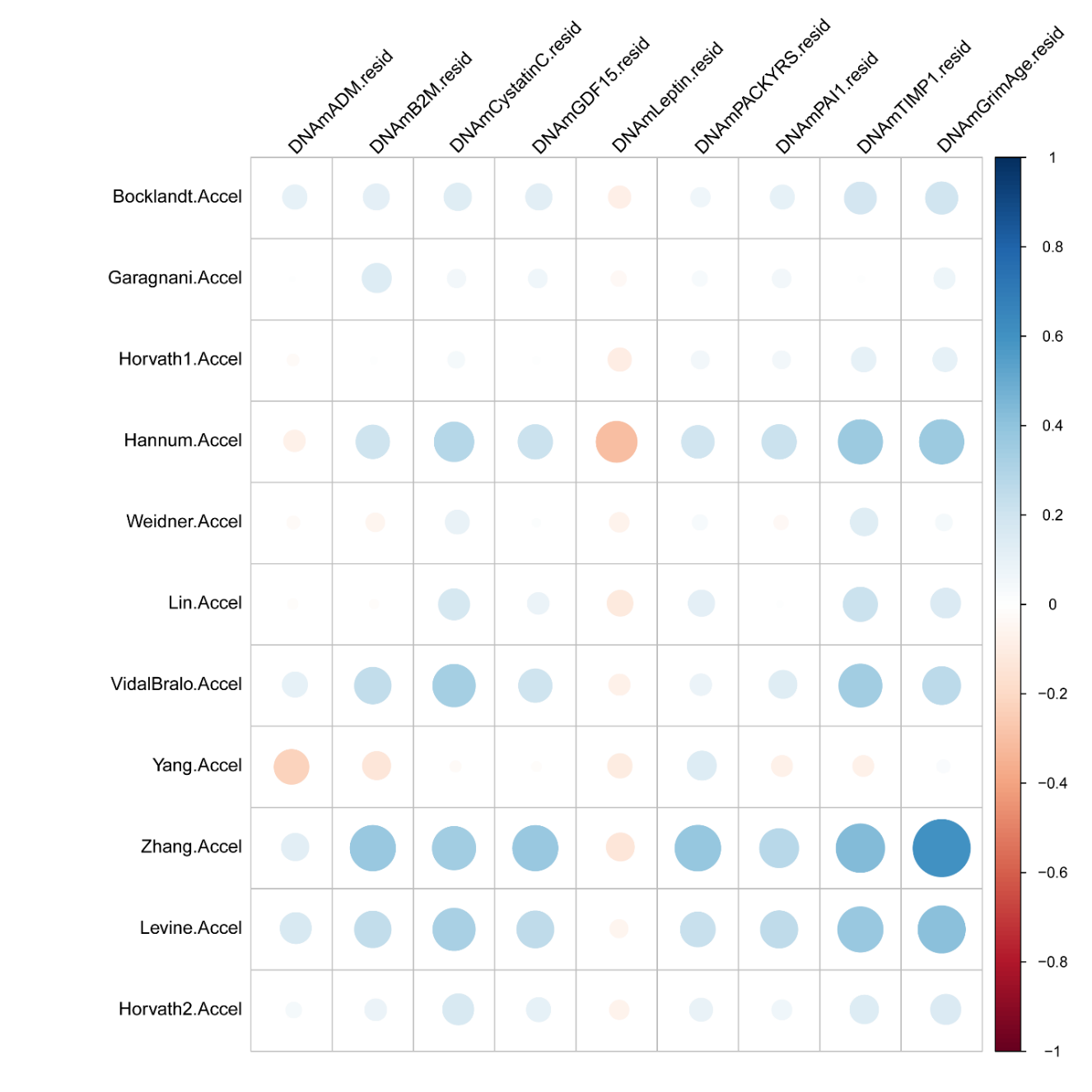


**Figure S14. Correlation between clock accelerations and GrimAge sub-measures after adjusting for chronological age.**

**Figure S15. Distributions of modules represented across CpGs in each clock.** In comparing the proportions of consensus co-methylation modules across the various clocks, we find that Hannum has a significantly higher proportion of CpGs in the brown module, and both Hannum and Lin are further enriched for CpGs in the yellow module. Surprisingly Horvath1 and Levine had very similar proportions and were enriched for CpGs in the turquoise module, which was almost entirely absent from any of the other clocks.

**Figure S16. Heritability estimates of the eleven epigenetic clocks.** Bar heights represent SNP-based heritability estimates calculated using GCTA. Error bars represent standard errors. Blue bars denote heritability estimates at or above h2=0.20, whereas red bars denote heritability estimates below 0.20.

## ***Heritability estimates of the eleven epigenetic clocks***

Using data (n=3684) from Framingham Heart Study (FHS), we estimated the variability in each clock explained by all SNPs while controlling for age, sex, and the first four principal components of genetic ancestry. We found that the six clocks (Hannum, Lin, Yang, Levine, Horvath1, and Horvath2) had relatively low heritability with values ranging from 0.10 (Horvath1, Figure S16) to 0.18 (Yang). Whereas the other five clocks had higher heritability, with the highest for Bocklandt (0.34). Complete results are available in Table S10.

**EXPERIMENTAL PROCEDURES**

## **DATA**

The majority of the data used for this study is publicly available via Gene Expression Omnibus (GEO). Details for each data source is provided below. Briefly, for multi-tissue analysis, we used Illumina Infinium 450k DNA methylation data from: breast (GSE101961), buccal (GSE94876), cerebellum (part of GSE89706), colon (GSE101764), dermis (part of GSE51954), dorsolateral prefrontal cortex (DLPFX, GSE74193), epidermis (part of GSE51954), fibroblasts (GSE77135), frontal cortex bulk (part of GSE66351), hippocampus (part of GSE89706), purified monocytes (GSE56046), occipital cortex glia (part of GSE66351), occipital cortex neurons (part of GSE66351), striatum (part of GSE89706), temporal cortex bulk (part of GSE66351), and whole blood (GSE87571). For transcriptomics analysis, we used gene expression data from: monocytes (GSE56045) and DLPFC (AMP-AD Knowledge Portal). For analysis involving mitochondria, cell senescence, and cancer, we used Illumina Infinium 450k DNA methylation data including GSE100249, GSE91069, and GSE53051, respectively. For proteomics analysis, we used DNA methylation and proteomic data from Genetic and Epigenetic Signatures of Translational Aging Laboratory Testing (GESTALT), mentioned elsewhere (Tanaka et al., 2018). To develop the ‘meta-clock’, we used DNA methylation data from Framingham Heart Study (FHS, Offspring Cohort).

**Breast (GSE101961):** This dataset measured DNA methylation in the normal breast tissues using the Illumina Infinium 450K Human Methylation Beadchip. It consists of 121 cancer-free women (mean age: 38 years, range: 17-76 years), provided by Song et al (Song et al., 2017).

**Buccal (GSE94876):** This dataset measured DNA methylation in the buccal cells using the Illumina Infinium 450K Human Methylation Beadchip. It includes 120 generally healthy adult males (mean age: 38 years, range: 17-76 years) from the smokers (SMK), moist snuff consumers (MSC), and Non-Tobacco Consumers (NTC) cohorts (40 participants/cohort). It is provided by Jessen et al (Jessen, Borgerding, & Prasad, 2018).

**Cerebellum (part of GSE89706):** This dataset measured DNA methylation in the post-mortem brain samples, representing tissue from four brain regions (prefrontal cortex, striatum, hippocampus and cerebellum), using the Illumina Infinium 450K Human Methylation Beadchip. It includes 262 samples from 41 schizophrenia patients and 47 controls. Here we used the control cerebellum subsamples (n=33, mean age: 45.12 years, range: 21-72 years). This dataset is provided by Viana et al (Viana et al., 2017).

**Colon (GSE101764) :** This dataset measured DNA methylation in 149 mucosa and 112 colorectal cancer tissues using the Illumina Infinium 450K Human Methylation Beadchip. In this study, we used the 149 mucosa colorectal mucosa samples (mean age: 63 years, range: 22-89 years). This dataset was provided by Barrow et al (Barrow et al., 2017).

**Dermis (part of GSE51954):** This dataset measured DNA methylation in the dermal and epidermal samples using the Illumina Infinium 450K Human Methylation Beadchip. It consists of 40 dermal and 38 epidermal samples. Here we used the dermis subsample (mean age: 50 years, range: 20-90 years). This dataset is provided by Vandiver et al (Vandiver et al., 2015).

**Dorsolateral prefrontal cortex (DLPFC, GSE74193):** This dataset measured DNA in the dorsolateral prefrontal cortex brain tissue using the Illumina Infinium 450K Human Methylation Beadchip. It consists of 675 samples (mean age: 35 years, range: -0.5-97 years), provided by Jaffe et al (Jaffe et al., 2016).

**Epidermis (part of GSE51954):** This dataset has been described above. Here we used the 38 epidermal samples (mean age: 51 years, range: 20-90 years).

**Fibroblasts (GSE77135) :** This dataset measured in postmortem dural and scalp fibroblasts using the Illumina Infinium 450K Human Methylation Beadchip. It consists of 21 samples (11 intrinsically matched pairs of dural and scalp fibroblasts, 1 was removed in preprocessing) (mean age: 31.7 years, range: 0-85 years), and is provided by Ivanov et al (Ivanov et al., 2016).

**Frontal Cortex bulk (part of GSE66351):** This dataset measured in postmortem human brains using the Illumina Infinium 450K Human Methylation Beadchip. It consists of 63 samples from bulk cells in the frontal cortex (mean age: 73.9 years, range: 18-97 years). This dataset is provided by Gasparoni et al (Gasparoni et al., 2018).

**Hippocampus (part of GSE89706):** This dataset has been described above. Here we used the control Hippocampus subsamples (n=27, mean age: 62 years, range: 25-95 years).

**Monocytes (GSE56046) :** This dataset measured DNA methylation in purified monocytes using the Illumina Infinium 450K Human Methylation Beadchip. These samples were from 1,202 participants from the Multi-Ethnic Study of Atherosclerosis (MESA) study (Reynolds et al., 2015). As both the methylation and transcriptomic (GSE56045) datasets from purified monocytes from these participants were used in this study, we briefly described the MESA study. The MESA is a population cohort study with the aim of examining the prevalence, correlates, and progression of subclinical cardiovascular disease since 2000. Data on socio-demographic, lifestyles, nutrition, laboratory, extensive clinical, and medication were collected by five clinic visits. The sample used for transcriptome and methylation analysis were from April 2010 to February 2012 examination (Exam 5) of 1,264 randomly selected participants from four MESA field centers (Baltimore, MD; Forsyth County, NC; New York, NY; and St. Paul,MN) (Reynolds et al., 2015). The details on blood specimen collections, purification of CD14+ monocytes, DNA/RNA extraction, global expression quantification, epigenome-wide methylation quantification, quality control and pre-processing of microarray data were provided elsewhere (Reynolds et al., 2015). Regarding participants included in the methylation and transcriptomic datasets, their mean age was 60 years, with the range of 44 to 83 years. This dataset also includes covariates such as race, gender, study site, and residual sample contamination (i.e., separate enrichment scores for neutrophils, B cells, T cells, and natural killer cells) for further monocyte data analysis. This dataset was provided by Reynolds et al (Reynolds et al., 2015). Microarray transcriptomic data was also available based on Illumina HumanHT-12 v4 Expression BeadChip.

**Occipital Cortex Glia and Neurons (part of GSE66351) :** This dataset has been described above. Here we used the sorted cells (glia and neurons) from the occipital cortex (n=31 each cell type, mean age: 74.77 years, range: 18-94 years).

**Striatum (part of GSE89706):** This dataset has been described above. Here we used the control Striatum (putamen) subsamples (n=82, mean age: years, range: years).

**Temporal cortex bulk (part of GSE66351)**

This dataset has been described above. Here we used the Temporal cortex bulk subsamples (n=?, mean age: 56 years, range: 21-96 years).

**Datasets for mitochondrial analysis (GSE100249):** This dataset was used to explore the role of loss of mitochondrial DNA (mtDNA) in locus-specific alterations in histone acetylation, DNA methylation and expression of a number of genes. The mtDNA of HEK293 cells was depleted by doxycycline addition to the medium in order to turn a dominant negative mitochondrial DNA polymerase (DN-POLG). Samples were originally analyzed at days 0, 3, 6 and 9 for a total of four samples. There were three independent biological replicates at each time point and samples at time point 0 was used as controls. For methylation, nuclear DNA methylation was evaluated in 143B cells in which mtDNA was chronically depleted based on ethidium bromide treatment. Samples include three independent biological replicates of controls (rho+) and three of mtDNA depleted (rho0). This dataset was provided by Lozoya et al (Lozoya et al., 2018).

**Datasets for cell senescence (GSE91069):** This dataset measured DNA methylation for several types of cells using the Illumina Infinium 450K Human Methylation Beadchip. Details were provided elsewhere (Xie et al., 2018). Briefly, early passage (EP) or near-senescent (NS) human foreskin fibroblast BJ cells were infected with retrovirus packaged with pBabe-hygro-hTERT, pBabe-zeo-SV40 large T genomic DNA (LT and ST antigens) and pBabe-puro-HrasV12 to establish the immortalization-transformation lineage. These EP BJ cells were also infected with mock retrovirus as a control and were cultured accordingly with the transformation processes. When the authors got the early transformed cells, early passage BJ cells roughly underwent 14 population doublings and become near-senescent. These near-senescent cells were continuously cultured until they ceased to proliferate and fully senesced. The authors also got oncogene-induced senescence (OIS) when EP BJ cells were infected with retrovirus encoding HrasV12. This dataset was provided by Xie et al (Xie et al., 2018).

**Datasets for cancer (GSE53051)**: This dataset examined methylation status using HumanMethylation450 BeadChip for samples from 5 different tumor types (breast, colon, lung, pancreas, and thyroid cancer), some of which have early stage samples. In this study, we only used the data for normal and cancer samples. This dataset was provided by Timp et al (Timp et al., 2014).

**Framingham Heart Study (FHS): Offspring and Third Generation Cohorts:** In the last step of this study, we used DNA methylation data from FHS, involving two cohorts: the FHS offspring cohort and the Third-generation cohort. Detailed descriptions of these cohorts have been published (Kannel, Feinleib, McNamara, Garrison, & Castelli, 1979; Splansky et al., 2007; Tsao & Vasan, 2015). In brief, the FHS offspring cohort began enrollment in 1971 and included 5,124 offspring of the FHS original cohort. Around 2800 participants attended the eighth examination cycle (2005-2008) and consented to provide their DNA to be used for genomic research. The adult children (third generation cohort, N=4,095) of the offspring cohort were recruited and examined between 2002-2005. Around 1500 participants of the third-generation cohort attended the second exam cycle (2005-2008) and consented to provide their DNA to be used for genomic research. All participants provided written informed consent at the time of each examination visit. The study protocol was approved by the Institutional Review Board at Boston University Medical Center (Boston, MA). DNA methylation, assayed with the Infinium HumanMethylation450 BeadChip, was available for 4149 participants for the current study (available in dbGaP, accession number: phs000724.v7.p11). Deaths of the FHS participants that occurred prior to January 1, 2014 were ascertained using multiple strategies, including routine contact with participants for health history updates, surveillance at the local hospital and in obituaries of the local newspaper, and queries to the National Death Index. Death certificates, hospital and nursing home records prior to death, and autopsy reports were requested. When cause of death was undeterminable, the next of kin were interviewed. The date and cause of death were reviewed by an endpoint panel of 3 investigators.

**ROSMAP DLPFC data:** DNAm data measured via using the Illumina Infinium 450K Human Methylation Beadchip, and microarray transcriptomic data measured via the Illumina HumanHT-12 Expression BeadChip from n=718 DLPFC samples was analyzed. The samples were from the Religious Order Study (ROS) and the Memory and Aging Project (MAP), both of which are longitudinal community-based cohort studies of aging and dementia (Bennett, Schneider, Arvanitakis, & Wilson, 2012; Bennett et al., 2005). The ROS was initialed in 1994 and enrolled individuals from religious communities for longitudinal clinical analysis and brain donation. Enrollment required no known signed of dementia. Alzheimer's Disease (AD) status was determined by a computer algorithm based on cognitive test performance with a series of discrete clinical judgments made in series by a neuropsychologist and a clinician. In 1997, the MAP was launched to complement the ROS by enrolling individuals with a wider range of life experiences and socioeconomic status into a study of similar structure and design as ROS. The MAP enrolled older individuals without any signs of dementia, primarily recruiting from continuous care retirement communities throughout northeastern Illinois, USA. It used the identical method to determine AD. The ROSMAP is run by Rush University. The procedures follow those outlined by the pathologic dataset recommended by the National Alzheimer’s Disease Coordinating Center and pathologic diagnoses of AD use NIA-Reagan and modified CERAD criteria, and the staging of neurofibrillary pathology uses Braak Staging. The transcriptomic data was available for 490 samples (can be found in AMP-AD Knowledge Portal, Synapse ID: syn3800853), described previously (B. Zhang et al., 2013).

**Epigenetic clocks**

Each of the eleven published clocks considered in this study (Additional file 1), was calculated in accordance with published methods (Bocklandt et al., 2011; Garagnani et al., 2012; Hannum et al., 2013; Horvath, 2013; Horvath et al., 2018; Levine et al., 2018; Lin & Wagner, 2015; Vidal-Bralo et al., 2016; Weidner et al., 2014; Yang et al., 2016; Y. Zhang et al., 2017). To simplify the description, we used the last name of the first author to refer to each clock. Most of these clocks were developed to predict chronological age in whole blood, with the number of included CpGs ranging from 3 to 513—the exception being the clocks by Bocklandt and Garagnani, which are each based on DNAm for only one CpG. In this study, we also calculated the age acceleration, defined as the residual resulting from a linear model when regressing epigenetic age on chronological age. As mentioned, the age acceleration is meant to reflect between-person and/or between-tissue variably in the rate of aging—whether a person appears older (positive value) or younger (negative value) than expected (Chen et al., 2016; Horvath, 2013; Horvath & Raj, 2018).

**Data Analyses**

The analytic plan is briefly described in Figure 1. To examine clock characteristics across tissues/cells, we first, we: 1) described the overlap in CpG sites and/or CpG blocks across the eleven clocks; 2) compared the CpG types/targets included in each of the clocks (e.g., the proportion of CpG in high density islands, PcG protein targets, DNase I hypersensitive sites); and 3) tested the age correlations and age-specific variance of CpGs within each clock, using data from multiple tissues and cell types, representing the full age range from fetal up to extreme old age.

Second, we calculated the eleven epigenetic clocks using a variety of tissues and cell types. We then examine their age correlations across pooled samples, as well as within six tissues/cells— monocytes, DLPFC, colon, fibroblasts, epidermis, glial from occipital cortex, neurons from occipital cortex.

Third, to gain additional insight into aging relationships, we examined epigenetic clock associations with a novel SOMA based plasma proteomic clock developed by Tanaka et al (Tanaka et al., 2018), using whole blood DNAm data from the GESTALT study. We also performed network analysis (weighted-gene correlation network analysis (WGCNA) (Langfelder & Horvath, 2008)) for all proteins and linked protein modules (PMs) to the various clocks. Briefly, for WGCNA, we designated a “signed” network, and employed a thresholding power (picked using pickSoftThreshold function, here 11) and dynamic tree cut. For each resultant module, WGCNA used using principle component analysis as default to estimate the eigengene value—representing the optimal summary of the protein (or gene) expression profile for that modules.

To investigate the functional (transcriptional) signatures of these clocks, we related epigenetic clocks to transcriptomic data. Using data from purified monocytes (GSE56046), we first identified genes (denoted as differentially expressed genes, DEGs) that were associated with age residuals for at least one of the eleven epigenetic clocks (FDR<0.05), using R package “limma” (Ritchie et al., 2015). We then compared the gene-specific log2FC values corresponding to eleven epigenetic clocks (age residuals), to determine if clocks exhibited shared transcriptomic signals. We ran similar analysis for gene expression data from brain (DLPFC). Next, we performed weighted-gene correlation network analysis (WGCNA) (Langfelder & Horvath, 2008) to identify co-expression modules for 8589 overlapped genes between monocytes and brain. For each module, we estimated the eigengene value—representing the optimal summary of the gene expression profile for gene assigned to that modules—and then related these module eigengenes to the epigenetic clock age residuals. We then performed functional enrichment analysis for Gene Ontology (GO) terms and KEGG pathways for the co-expression modules, using the R package “clusterProfiler” (Yu, Wang, Han, & He, 2012). We also used WebGestalt (<http://www.webgestalt.org/option.php>, a web-based pathway analysis tool) (Wang, Vasaikar, Shi, Greer, & Zhang, 2017) to validate our results (the default minimum pathway size (n=5) and significance level of FDR <0.05 were used). We presented the top five GO terms and KEGG pathways for four interesting modules.

For functional annotation analysis (enrichment analysis for Gene Ontology (GO) terms and KEGG pathways), we mainly used the R package “clusterProfiler” (Yu et al., 2012) as mentioned in the text. However, we also used WebGestalt (<http://www.webgestalt.org/option.php>, a web-based pathway analysis tool) (Wang et al., 2017) to validate our results. We used the default minimum pathway size (n=5) and significance level of FDR <0.05.

To provide evidence linking epigenetic clocks and two well-known hallmarks of aging—cellular senescence, and mitochondrial depletion—we examined DNAm data from cultured fibroblasts and 143B cells. For instance, epigenetic clock scores for Human BJ Fibroblasts undergoing either replicative senescence or oncogene induced senescence (GSE91069) were compared to early passage cells. We then examined the relationship between mitochondrial DNA (mtDNA) depletion and epigenetic aging using in vitro DNAm data from 143B cells (GSE100249) [30]. Clock scores were estimated and then compared between 143B cells with chronically depleted mDNA (rho0) and 143B controls.

Given the link between epigenetics and cancer (Ambatipudi et al., 2017; Levine et al., 2015; Teschendorff et al., 2010; Yang et al., 2016; Zheng et al., 2016), we examined how these epigenetic clocks differ in terms of distinguishing tumor vs. normal tissues using a dataset (GSE53051) that included five different tumor types (breast, colon, lung, pancreas, and thyroid cancer) (Timp et al., 2014). We focused on the six epigenetic clocks that have been shown to have conserved signals in above analysis. Comparisons between tumor versus normal samples were assessed after adjusting for the age of the donor and the tissue type.

We performed heritability analysis of the eleven epigenetic clocks using data on a large sample (N=3684) from Framingham Heart Study (FHS). When calculating the SNP-based heritability, we controlled for age, sex, and the first four principal components of genetic ancestry. Given that bivariate analysis is not recommended for the family data and models including only a subset of unrelated samples did not converge, we were unable to evaluate the genetic correlation between these clocks.

Finally, considering both shared and unique signals across the epigenetic clocks, we applied consensus WGCNA to identify co-methylation modules across the 1600 clock CpGs. We then used these modules to calculate submodule clocks, such that we utilized the original clock equations, but restricted the input to one module at a time. For instance, the turquoise submodule for Horvath1 represents the portion of Horvath1 accounted for by the CpGs in the turquoise module. Thus, summing across the modules, would give you the overall clock score. These submodules (n=85 across all modules and all clocks) were then used to train a mortality predictor using DNAm in whole blood from the FHS. To accomplish this, we randomly divided 3,854 samples from the FHS into two groups—training (n=2911) and test (n=943). An elastic net cox proportional hazard model was used to fit a predictor of all-cause mortality in the training set and 14 submodule clocks were selected (Table S7). The resulting score was then estimated in the test set and mortality prediction was contrasted against the two clocks in our study that have been shown to be robust predictors of remaining life expectancy (Levine and Zhang). We the estimated this score in the multi-tissue and in vitro data and tested for associations with age across tissues, aging hallmarks, tumorigenesis, and neuropathology.

**Heritability Analysis**

*Framingham Heart Study Data Preparation*

Framingham Heart Study data from the Child Consent Set phs000342 v17.p10.c1 was used for this analysis. Genotype data was imputed to 1000 Genomes (November 2010 release) and included 6834 individuals. MACH output files for each of the 22 autosomes were first converted to PLINK format using the --dosage-mach-gz and --make-bed functions in GCTA (version 1.92.2). Pedigree information was added to each PLINK file, and each was filtered in PLINK (version 1.07) to remove SNPs with MAF<0.05, HWE p<=0.0001, and r^2^<=0.8.

*Creation of GRMs*

A genetic relationship matrix for each chromosome was created in GCTA with the --make-grm function. Because the order of subjects was slightly different for some of the chromosome files, the --grm-unify function was used to create a new set of GRMs with identical order. Each chromosomal GRM was then combined into a single GRM using the --mgrm function. Because of the family structure within the Framingham Heart Study, a second GRM was created from the combined one using the --make-bK function. This function employs the method proposed by Zaitlen et al (Zaitlen et al., 2013) and is described in the GCTA documentation (<https://cnsgenomics.com/software/gcta/#GREMLinfamilydata>). This method allows the estimation of SNP-based heritability in family data without removing related individuals. A threshold of 0.05 was used, which sets each element of the GRM less than 0.05 to zero.

*Heritability Estimation*

To estimate the variability explained by all SNPs used in the GRMs, restricted maximum likelihood (REML) analysis was performed in GCTA with the --reml function. The heritability was estimated for each of the 11 DNAm clocks controlling for age, sex, and the first 4 principal components.

This analysis included a total of 3684 individuals from 862 families that included those with clock phenotypes and genetic data. A summary of the results is included in the file clock_h2_summary.xlsx. This file includes estimates for V(G1)/Vp (the SNP-based heritability), the sum of V(G)/Vp (the pedigree-based heritability), and V(G2)/Vp (the difference between pedigree and SNP-based heritability). P-values for these were calculated using the pnorm function in R (version 3.5.1) using z=estimate/SE.

**References**

Ambatipudi, S., Horvath, S., Perrier, F., Cuenin, C., Hernandez-Vargas, H., Le Calvez-Kelm, F., . . . Herceg, Z. (2017). DNA methylome analysis identifies accelerated epigenetic ageing associated with postmenopausal breast cancer susceptibility. *European Journal of Cancer, 75*, 299-307. doi: <https://doi.org/10.1016/j.ejca.2017.01.014>

Barrow, T. M., Klett, H., Toth, R., Bohm, J., Gigic, B., Habermann, N., . . . Michels, K. B. (2017). Smoking is associated with hypermethylation of the APC 1A promoter in colorectal cancer: the ColoCare Study. *J Pathol, 243*(3), 366-375. doi: 10.1002/path.4955

Bennett, D. A., Schneider, J. A., Arvanitakis, Z., & Wilson, R. S. (2012). Overview and findings from the religious orders study. *Curr Alzheimer Res, 9*(6), 628-645.

Bennett, D. A., Schneider, J. A., Buchman, A. S., Mendes de Leon, C., Bienias, J. L., & Wilson, R. S. (2005). The Rush Memory and Aging Project: study design and baseline characteristics of the study cohort. *Neuroepidemiology, 25*(4), 163-175. doi: 10.1159/000087446

Bocklandt, S., Lin, W., Sehl, M. E., Sanchez, F. J., Sinsheimer, J. S., Horvath, S., & Vilain, E. (2011). Epigenetic predictor of age. *PLoS One., 6*. doi: 10.1371/journal.pone.0014821

Chen, B. H., Marioni, R. E., Colicino, E., Peters, M. J., Ward-Caviness, C. K., Tsai, P. C., . . . Horvath, S. (2016). DNA methylation-based measures of biological age: meta-analysis predicting time to death. *Aging (Albany NY), 8*(9), 1844-1865. doi: 10.18632/aging.101020

Garagnani, P., Bacalini, M. G., Pirazzini, C., Gori, D., Giuliani, C., Mari, D., . . . Franceschi, C. (2012). Methylation of ELOVL2 gene as a new epigenetic marker of age. *Aging Cell, 11*(6), 1132-1134. doi: 10.1111/acel.12005

Gasparoni, G., Bultmann, S., Lutsik, P., Kraus, T. F. J., Sordon, S., Vlcek, J., . . . Walter, J. (2018). DNA methylation analysis on purified neurons and glia dissects age and Alzheimer's disease-specific changes in the human cortex. *Epigenetics Chromatin, 11*(1), 41. doi: 10.1186/s13072-018-0211-3

Hannum, G., Guinney, J., Zhao, L., Zhang, L., Hughes, G., Sadda, S., . . . Zhang, K. (2013). Genome-wide methylation profiles reveal quantitative views of human aging rates. *Mol Cell, 49*(2), 359-367. doi: 10.1016/j.molcel.2012.10.016

Horvath, S. (2013). DNA methylation age of human tissues and cell types. *Genome Biol, 14*(10), R115. doi: 10.1186/gb-2013-14-10-r115

Horvath, S., Oshima, J., Martin, G. M., Lu, A. T., Quach, A., Cohen, H., . . . Raj, K. (2018). Epigenetic clock for skin and blood cells applied to Hutchinson Gilford Progeria Syndrome and ex vivo studies. *Aging (Albany NY), 10*(7), 1758-1775. doi: 10.18632/aging.101508

Horvath, S., & Raj, K. (2018). DNA methylation-based biomarkers and the epigenetic clock theory of ageing. *Nat Rev Genet, 19*(6), 371-384. doi: 10.1038/s41576-018-0004-3

Ivanov, N. A., Tao, R., Chenoweth, J. G., Brandtjen, A., Mighdoll, M. I., Genova, J. D., . . . Jaffe, A. E. (2016). Strong Components of Epigenetic Memory in Cultured Human Fibroblasts Related to Site of Origin and Donor Age. *PLoS Genet, 12*(2), e1005819. doi: 10.1371/journal.pgen.1005819

Jaffe, A. E., Gao, Y., Deep-Soboslay, A., Tao, R., Hyde, T. M., Weinberger, D. R., & Kleinman, J. E. (2016). Mapping DNA methylation across development, genotype and schizophrenia in the human frontal cortex. *Nat Neurosci, 19*(1), 40-47. doi: 10.1038/nn.4181

Jessen, W. J., Borgerding, M. F., & Prasad, G. L. (2018). Global methylation profiles in buccal cells of long-term smokers and moist snuff consumers. *Biomarkers, 23*(7), 625-639. doi: 10.1080/1354750X.2018.1466367

Kannel, W. B., Feinleib, M., McNamara, P. M., Garrison, R. J., & Castelli, W. P. (1979). An investigation of coronary heart disease in families. The Framingham offspring study. *Am J Epidemiol, 110*(3), 281-290. doi: 10.1093/oxfordjournals.aje.a112813

Langfelder, P., & Horvath, S. (2008). WGCNA: an R package for weighted correlation network analysis. *BMC Bioinformatics, 9*, 559. doi: 10.1186/1471-2105-9-559

Levine, M. E., Hosgood, H. D., Chen, B., Absher, D., Assimes, T., & Horvath, S. (2015). DNA methylation age of blood predicts future onset of lung cancer in the women's health initiative. *Aging (Albany NY), 7*(9), 690-700. doi: 10.18632/aging.100809

Levine, M. E., Lu, A. T., Quach, A., Chen, B. H., Assimes, T. L., Bandinelli, S., . . . Horvath, S. (2018). An epigenetic biomarker of aging for lifespan and healthspan. *Aging (Albany NY), 10*(4), 573-591. doi: 10.18632/aging.101414

Lin, Q., & Wagner, W. (2015). Epigenetic Aging Signatures Are Coherently Modified in Cancer. *PLoS Genet, 11*(6), e1005334. doi: 10.1371/journal.pgen.1005334

Liu, Z., Kuo, P.-L., Horvath, S., Crimmins, E., Ferrucci, L., & Levine, M. (2018). Phenotypic Age: a novel signature of mortality and morbidity risk. *bioRxiv*. doi: 10.1101/363291

Lozoya, O. A., Martinez-Reyes, I., Wang, T., Grenet, D., Bushel, P., Li, J., . . . Santos, J. H. (2018). Mitochondrial nicotinamide adenine dinucleotide reduced (NADH) oxidation links the tricarboxylic acid (TCA) cycle with methionine metabolism and nuclear DNA methylation. *PLoS Biol, 16*(4), e2005707. doi: 10.1371/journal.pbio.2005707

Reynolds, L. M., Ding, J., Taylor, J. R., Lohman, K., Soranzo, N., de la Fuente, A., . . . Liu, Y. (2015). Transcriptomic profiles of aging in purified human immune cells. *BMC Genomics, 16*, 333. doi: 10.1186/s12864-015-1522-4

Ritchie, M. E., Phipson, B., Wu, D., Hu, Y., Law, C. W., Shi, W., & Smyth, G. K. (2015). limma powers differential expression analyses for RNA-sequencing and microarray studies. *Nucleic Acids Res, 43*(7), e47. doi: 10.1093/nar/gkv007

Song, M. A., Brasky, T. M., Weng, D. Y., McElroy, J. P., Marian, C., Higgins, M. J., . . . Shields, P. G. (2017). Landscape of genome-wide age-related DNA methylation in breast tissue. *Oncotarget, 8*(70), 114648-114662. doi: 10.18632/oncotarget.22754

Splansky, G. L., Corey, D., Yang, Q., Atwood, L. D., Cupples, L. A., Benjamin, E. J., . . . Levy, D. (2007). The Third Generation Cohort of the National Heart, Lung, and Blood Institute's Framingham Heart Study: design, recruitment, and initial examination. *Am J Epidemiol, 165*(11), 1328-1335. doi: 10.1093/aje/kwm021

Tanaka, T., Biancotto, A., Moaddel, R., Moore, A. Z., Gonzalez-Freire, M., Aon, M. A., . . . Ferrucci, L. (2018). Plasma proteomic signature of age in healthy humans. *Aging Cell, 17*(5), e12799. doi: 10.1111/acel.12799

Teschendorff, A. E., Menon, U., Gentry-Maharaj, A., Ramus, S. J., Weisenberger, D. J., Shen, H., . . . Maxwell, A. P. (2010). Age-dependent DNA methylation of genes that are suppressed in stem cells is a hallmark of cancer. *Genome research, 20*(4), 440-446.

Timp, W., Bravo, H. C., McDonald, O. G., Goggins, M., Umbricht, C., Zeiger, M., . . . Irizarry, R. A. (2014). Large hypomethylated blocks as a universal defining epigenetic alteration in human solid tumors. *Genome Med, 6*(8), 61. doi: 10.1186/s13073-014-0061-y

Tsao, C. W., & Vasan, R. S. (2015). Cohort Profile: The Framingham Heart Study (FHS): overview of milestones in cardiovascular epidemiology. *Int J Epidemiol, 44*(6), 1800-1813. doi: 10.1093/ije/dyv337

Vandiver, A. R., Irizarry, R. A., Hansen, K. D., Garza, L. A., Runarsson, A., Li, X., . . . Feinberg, A. P. (2015). Age and sun exposure-related widespread genomic blocks of hypomethylation in nonmalignant skin. *Genome Biol, 16*, 80. doi: 10.1186/s13059-015-0644-y

Viana, J., Hannon, E., Dempster, E., Pidsley, R., Macdonald, R., Knox, O., . . . Mill, J. (2017). Schizophrenia-associated methylomic variation: molecular signatures of disease and polygenic risk burden across multiple brain regions. *Hum Mol Genet, 26*(1), 210-225. doi: 10.1093/hmg/ddw373

Vidal-Bralo, L., Lopez-Golan, Y., & Gonzalez, A. (2016). Simplified Assay for Epigenetic Age Estimation in Whole Blood of Adults. *Frontiers in genetics, 7*, 126-126. doi: 10.3389/fgene.2016.00126

Wang, J., Vasaikar, S., Shi, Z., Greer, M., & Zhang, B. (2017). WebGestalt 2017: a more comprehensive, powerful, flexible and interactive gene set enrichment analysis toolkit. *Nucleic Acids Res, 45*(W1), W130-W137. doi: 10.1093/nar/gkx356

Weidner, C. I., Lin, Q., Koch, C. M., Eisele, L., Beier, F., Ziegler, P., . . . Wagner, W. (2014). Aging of blood can be tracked by DNA methylation changes at just three CpG sites. *Genome Biology, 15*(2), R24. doi: 10.1186/gb-2014-15-2-r24

Xie, W., Kagiampakis, I., Pan, L., Zhang, Y. W., Murphy, L., Tao, Y., . . . Easwaran, H. (2018). DNA Methylation Patterns Separate Senescence from Transformation Potential and Indicate Cancer Risk. *Cancer Cell, 33*(2), 309-321 e305. doi: 10.1016/j.ccell.2018.01.008

Yang, Z., Wong, A., Kuh, D., Paul, D. S., Rakyan, V. K., Leslie, R. D., . . . Teschendorff, A. E. (2016). Correlation of an epigenetic mitotic clock with cancer risk. *Genome Biol, 17*(1), 205. doi: 10.1186/s13059-016-1064-3

Yu, G., Wang, L. G., Han, Y., & He, Q. Y. (2012). clusterProfiler: an R package for comparing biological themes among gene clusters. *OMICS, 16*(5), 284-287. doi: 10.1089/omi.2011.0118

Zaitlen, N., Kraft, P., Patterson, N., Pasaniuc, B., Bhatia, G., Pollack, S., & Price, A. L. (2013). Using extended genealogy to estimate components of heritability for 23 quantitative and dichotomous traits. *PLoS Genet, 9*(5), e1003520. doi: 10.1371/journal.pgen.1003520

Zhang, B., Gaiteri, C., Bodea, L. G., Wang, Z., McElwee, J., Podtelezhnikov, A. A., . . . Emilsson, V. (2013). Integrated systems approach identifies genetic nodes and networks in late-onset Alzheimer's disease. *Cell, 153*(3), 707-720. doi: 10.1016/j.cell.2013.03.030

Zhang, Y., Wilson, R., Heiss, J., Breitling, L. P., Saum, K. U., Schottker, B., . . . Brenner, H. (2017). DNA methylation signatures in peripheral blood strongly predict all-cause mortality. *Nat Commun, 8*, 14617. doi: 10.1038/ncomms14617

Zheng, Y., Joyce, B. T., Colicino, E., Liu, L., Zhang, W., Dai, Q., . . . Hou, L. (2016). Blood Epigenetic Age may Predict Cancer Incidence and Mortality. *EBioMedicine, 5*, 68-73. doi: 10.1016/j.ebiom.2016.02.008
